# Building trait datasets: effect of methodological choice on a study of invasion

**DOI:** 10.1007/s00442-022-05230-8

**Published:** 2022-08-17

**Authors:** Estibaliz Palma, Peter A. Vesk, Jane A. Catford

**Affiliations:** 1 School of Ecosystem and Forest Sciences, The University of Melbourne, Parkville, VIC 3010 Australia; 2 Department of Geography, King’s College London, 30 Aldwych, London, WC2B 4BG UK

**Keywords:** Exotic, Functional traits, Invasive, Plants, TRY

## Abstract

Trait-based approaches are commonly used to understand ecological phenomena and processes. Trait data are typically gathered by measuring local specimens, retrieving published records, or a combination of the two. Implications of methodological choices in trait-based ecological studies—including source of data, imputation technique, and species selection criteria—are poorly understood. We ask: do different approaches for dataset-building lead to meaningful differences in trait datasets? If so, do these differences influence findings of a trait-based examination of plant invasiveness, measured as abundance and spread rate? We collected on-site (Victoria, Australia) and off-site (TRY database) height and specific leaf area records for as many species as possible out of 157 exotic herbaceous plants. For each trait, we built six datasets of species-level means using records collected on-site, off-site, on-site and off-site combined, and off-site supplemented via imputation based on phylogeny and/or trait correlations. For both traits, the six datasets were weakly correlated (*ρ* = 0.31–0.95 for height; *ρ* = 0.14–0.88 for SLA), reflecting differences in species’ trait values from the various estimations. Inconsistencies in species’ trait means across datasets did not translate into large differences in trait-invasion relationships. Although we did not find that methodological choices for building trait datasets greatly affected ecological inference about local invasion processes, we nevertheless recommend: (1) using on-site records to answer local-scale ecological questions whenever possible, and (2) transparency around methodological decisions related to selection of study species and estimation of missing trait values.

**Supplementary Information:**

The online version contains supplementary material available at 10.1007/s00442-022-05230-8.

## Introduction

Trait-based approaches are commonly used to understand drivers of community assembly and environmental change—including biological invasions—as they provide a link between species’ population performance and their surrounding environment (Godoy et al. 2012; Lai et al. 2015; Carboni et al. 2016; Catford et al. 2020). Together with trait selection, how trait datasets are built (source of trait records, treatment of missing trait values, etc.) could be crucially important for ecological inference; the approach for collecting trait data may influence the resulting trait dataset (e.g. how trait variability is captured) and, therefore, the findings of a study (Lavorel et al. 2008; Kim et al. 2018). Unfortunately, this issue of dataset assembly is often overlooked and there is little information on how approaches for building trait datasets may affect ecological inference (Violle et al. 2015a).

Trait datasets are built based on researchers’ expertise and resources, availability of existing data and the scope of the research question. Trait records are typically gathered by measuring locally collected specimens (on-site collection), retrieving previously published records from the literature and online databases (off-site collection; e.g. from TRY, Kattge et al. (2020)) or a combination of the two. Locally sourced records sample populations that occur in the focal study region and thus may capture species’ adaptations to the local biotic and abiotic conditions (Cornwell and Ackerly 2009). Records sourced from global, online databases usually include measurements of populations occurring at any point over the species’ global geographic range, and thus may reflect species’ intraspecific variability at a larger scale rather than species’ local phenotypes (Table 1). The source of trait records may be particularly relevant for the study of biological invasions since species’ traits may differ between their native and introduced populations (van Kleunen et al. 2010a). Moreover, measurement methodology may vary significantly among the records within large databases, contributing to potential systematic and random errors in the trait data acquired.

**Table 1:** Summary of the six datasets examined in this study

Dataset	Ecological implications	Sampling size and trait mean estimation	Invasiveness question	Examples
***I)**** On-site data*	Trait records are collected locally. They are expected to encompass lower intraspecific variability than records collected at a global scale due to local adaptation (i.e. phenotypic plasticity or rapid adaptive evolution) and introduction bias (in the case of exotic species)	Collecting trait records across Victoria (230,000 km^2^), even with considerable sampling effort, may lead to small sample sizes for some species, especially for those with patchy occurrence or low abundance. Low sample size may translate into inaccurate estimation of trait mean	Are traits exhibited locally correlated with species’ ability to become locally invasive?	Jung et al. (2010) Lai et al. (2015)
*Off-site data and ****II)**** taxonomic, ****III)**** phylogenetic, or ****IV)**** bhpmf imputation*	Trait records are collected globally. They are expected to reflect species’ intraspecific trait variability over their global range. These records potentially reflect species’ adaptation and plasticity over environmental gradients larger than those found locally	Readily available trait records can potentially have uneven coverage of species, geographic areas and habitats, which may result in low sample size and (easily overlooked) poor representation for some species, e.g. most records for a given species coming from a low number of populations or similar environments Accuracy of imputation techniques is largely unknown	Are traits exhibited across the species’ global range (including both native and introduced areas) correlated with species’ ability to become locally invasive?	Lai et al. (2015) Schrodt et al. (2015) Palma et al. (2017) Swenson et al. (2017) Kim et al. (2018)
***V)**** On- & Off-site data*	Records are collected both locally and globally. Both sources are combined in a way that the local-scale trait variability is used to increase accuracy in the species’ trait variability across its global range	Use of existing knowledge about species’ global trait variability may reduce uncertainty linked to small sample sizes (due to limited geographic range) or poor representation (due to limited environmental heterogeneity) in local sampling. This approach may work best when: (1) the environmental conditions that species occupy at local and global scales are similar, and (2) the global trait records are collected in an unbiased manner throughout the species’ global distribution	Are traits exhibited locally correlated with species’ ability to become locally invasive?	Mitchell and Bakker (2014) Morris et al. (2015)
***VI)**** All off-site data*	Trait records are collected globally. They are expected to reflect species’ intraspecific trait variability over their global range, potentially reflecting species’ adaptation and plasticity over the whole environmental gradients they occupy	Inclusion of all species with available trait records (as opposed to defining study species based on other criteria) may enable a potentially larger number of species and higher statistical power in the associated analyses, and removes the need to impute trait means for species with missing values. However, issues related to uneven coverage of species, regions and habitats may still cause low sample size and poor representation for individual species. This type of dataset is likely biased towards species that are more abundant worldwide, i.e. those with larger geographic range and/or higher local abundance	Are traits exhibited across species’ global range (including both native and introduced areas) correlated to species’ ability to become invasive in a new range?	Adler et al. (2014) Gallagher and Leishman (2015)

*‘Ecological implications’* describes likely implications of acquiring trait data from species’ global distribution *vs* local and regional populations (we call these “local” for simplicity). *‘Sampling size and* t*rait mean estimation’* describes issues linked to sample sizes and implications for estimating trait means. *‘Invasiveness question’* states the ecological question that each dataset can address in our species invasiveness case study. *‘Examples’* lists studies that built trait datasets using an approach similar to the ones we examine. *bhpmf* stands for Hierarchical Bayesian Probabilistic Matrix Factorization

Trait-based studies often rely on species-level trait means (but see Godoy et al. (2012); Matzek (2012)), which may differ between on-site and off-site records if local conditions select for particular trait values that promote species’ fitness locally (e.g. taller individuals show higher survival rates in certain situations). From a methodological point of view, including trait records collected across a species’ full geographic range can increase number of replicates per species, potentially improving the accuracy of trait mean estimates (i.e. it will be closer to the ‘truth’) (Table 1). However, previously published trait information rarely covers all taxa of interest and sampling effort can be uneven across taxa and regions.

**Fig. 1:**
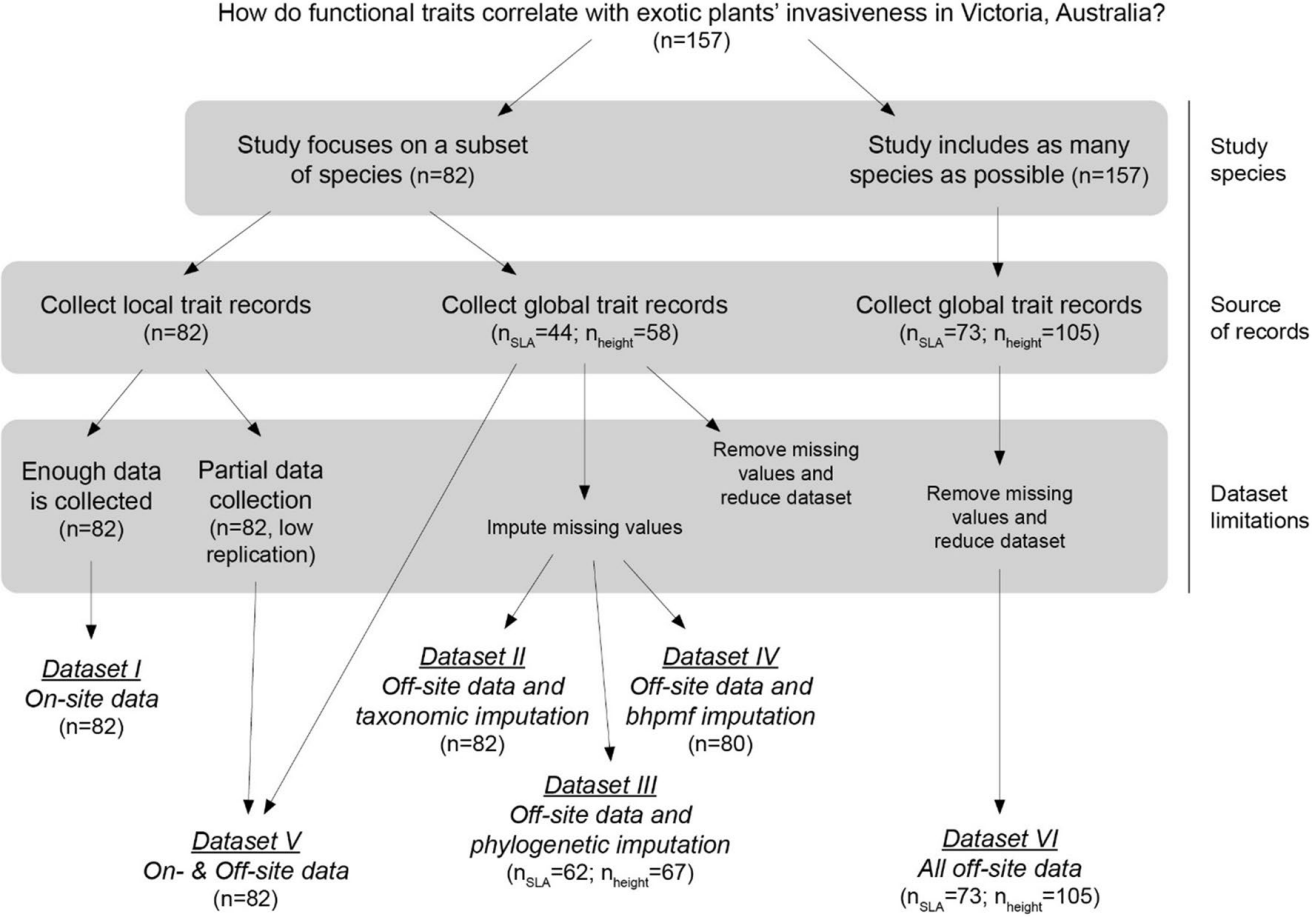
Six possible ways to build a trait dataset, based on selection criteria for the species of interest, the collection of local (on-site) *vs* global (off-site) trait records, and the approach used to handle missing information (see Methods). In our case study, we used these six datasets to answer the question *‘How do functional traits correlate to exotic plant species’ invasiveness in Victoria, Australia?’*. Sample size for each trait and step of the process for our Victoria case study is shown in parentheses. Out of the 157 exotic taxa representing independent invasiveness dimensions in Victoria, Australia, sample size across datasets is limited by the effectiveness of trait collection in Victoria, where 82 taxa were found, and/or by the availability of trait records in the TRY database. See Table S2 for the detailed list of species included in *Datasets I–VI*. *bhpmf* stands for Hierarchical Bayesian Probabilistic Matrix Factorization

Researchers deal with incomplete trait data in several ways (Fig. 1). Sometimes, they limit the dataset to only include species with available information. Broad scale studies of biogeographic patterns (e.g. Moles et al. (2014)) often avoid trait imputation by including as many species as possible with available trait data (Fig. 1), an approach that has also been used in regional scale studies (e.g. Speek et al. (2011)). Other times, researchers impute missing trait values through formal statistical techniques (Nakagawa and Freckleton 2008; Schrodt et al. 2015; Swenson et al. 2017), or informally using, e.g. means of congeneric or confamilial species that have trait records available (e.g. Catford et al. (2014)).

With the exception of imputation (Penone et al. 2014; Kim et al. 2018; Johnson et al. 2020), trait-based ecological studies rarely discuss implications of methodological choices required to build trait datasets (Violle et al. 2015a) despite the potential effect of methodological choice on analytical results. As with other subdisciplines in ecology, plant invasion studies have largely overlooked the influence that such methodological choices (namely source of trait records, imputation method, and study species selection) may have on the correlations between plant species’ traits and plant demographic success.

In this work, we examine six alternative approaches for building trait datasets of species-level means (Fig. 1), all of which are commonly used in ecological research (Table 1), and evaluate the effect of these choices on the relationship between plant traits and species invasiveness in the state of Victoria, southeast Australia. We use specific leaf area (SLA) and height as working examples and build six datasets for each trait. These datasets differ in: (i) the *source* of trait records—on-site (collected in Victoria) or off-site (collected across the whole geographic range of the species, as available in TRY database; Kattge et al. (2020)); (ii) the *imputation approach* to address missing or incomplete information; and (iii) the *study species selection criteri*a (Fig. 1). We first check whether on-site and off-site trait records are correlated, and how different methodological approaches modify species trait values and species’ rank order based on SLA and height means across the six datasets. We then focus on a case study of exotic plants in Victoria, Australia, and assess the relationship between species’ invasiveness (indicated by spread rate and local abundance; Catford et al. (2016); Palma et al. (2021a)) and traits. We discuss how methodological choices may influence conclusions drawn from trait-based invasiveness studies at regional levels.

## Materials and methods

### Plant species and compilation of trait datasets

We selected a group of 157 forb and grass species introduced to Victoria, Australia, based on a combination of stratified sampling of four independent invasiveness metrics (spread rate, local abundance, geographic range and environmental range; Catford et al. (2016)) and the availability of occurrence records—30 or more records available—from the Victorian Biodiversity Atlas (1970–2016; Victorian Department of Environment, Land, Water and Planning) and the Australasian Virtual Herbarium (1900–2016). Species’ invasiveness was assessed independently from their impact (Palma et al. 2021a). For as many of these species as possible, we gathered records of SLA and vegetative height, both from the online TRY database (Kattge et al. 2020) and in the field in Victoria, Australia (Oct–Dec 2015 and Sep–Nov 2016). Species names for these species, and all species used in this work as explained below, were standardised following The Plant List website (www.theplantlist.org) using R package *Taxonstand* (v 2.2).

Described below, we explored six possible approaches for building species-level mean trait datasets (Fig. 1), all of which are commonly used in ecological research (Table 1).

#### Dataset I—On-site data [*n* = 82]

We found wild populations for 82 out of the 157 exotic plant species in Victoria, Australia, and collected records of SLA and vegetative height from an average of 5 fully mature individuals grown in full sun for each species (Appendix S1). We collected a single sample for one species, and two samples for seven species because some of these species were very rare in Victoria and only 1–2 individuals were found. Where possible, we sampled individuals from different populations (Table S1, Fig. S1) to help capture intraspecific variation (see Appendix S1 for the correlation between species’ intraspecific trait variability and geographic distance between sampling locations).

#### Dataset II—Off-site data and taxonomic imputation [*n* = 82]

For the same 82 species included in *Dataset I (On-site data)*, we retrieved as many height and SLA records as possible through the TRY database (Kattge et al. 2020). For species without records in TRY, we imputed their trait values through a taxonomy-nested hierarchical model (family, genera and species are random effects), which borrowed information from all public SLA and height records from TRY:

TRAIT_t_ ~ Normal (mu_sp_t,s_, sd_obs_t_),

mu_sp_t,s_ ~ Normal (mu_g_t,g_, sd_sp_t,g_),

mu_g_t,g_ ~ Normal (mu_f_t,f_, sd_g_t_),

mu_f_t,f_ ~ Normal (mu_global_t_, sd_f_t_),

where TRAIT stands for each observation of trait t, which was log-transformed and standardised; mu_sp, mu_g and mu_f stand for the mean trait value for species s, genus g and family f, respectively; and sd_obs, sd_sp, sd_g and sd_f stand for the variability across observations, species, genera and families, respectively. To achieve good model convergence, the variability parameters were fixed, with the exception of sd_sp, which was allowed to vary among genera following an inverse gamma distribution. The models were built in R (R Core Team 2020) using a Bayesian inference framework through *R2jags* package (Su and Yajima 2015) (see Appendix S2 for coding details).

Of the 82 target species, we retrieved an average of 11 records of SLA for 44 species and 13 records of height for 58 species from TRY. We imputed SLA and height mean values for the remaining 38 and 24 species, respectively. Public records from TRY used for imputation included 12,594 observations of SLA (1621 species, 658 genera and 142 families) and 35,728 observations of height (4,190 species, 980 genera and 154 families). To reduce unwanted variation, we excluded records of woody species (our study species are all herbaceous), records collected under experimental or shady conditions, measurements from immature individuals, records from environmental conditions not found in Victoria (Appendix S3), and species only represented by a single record.

To evaluate the predictive power of this approach to estimate missing trait means, we used the same taxonomy-nested model described above to estimate the trait mean of the 44 and 58 species for which SLA and height records were available from TRY (but excluding them this time) and compared the imputed means with means drawn from their TRY records.

#### Dataset III—Off-site data and phylogenetic imputation [*n*_SLA_ = 62, *n*_height_ = 67]

As for *Dataset II (Off-site data and taxonomic imputation)*, we retrieved mean SLA and height for 44 and 58 species, respectively, from available records in TRY. Although not technically an imputation method, we increased the extent of the trait datasets by replacing species with missing values for *phylogenetic equivalent* species—i.e. the most phylogenetically similar species—for which public trait records were available in TRY. To identify these phylogenetic equivalents, we relied on R package *phyndr* (Pennell et al. 2016) and the phylogenetic tree published by Zanne et al. (2014). Imputed trait values (mean and standard deviation) for each species were estimated after 1,000 random iterations among the phylogenetic equivalents suggested by *phyndr* for each species. Phylogenetic equivalents with public TRY records of SLA and height were identified for 18 and 9 species, respectively, leading to a total of 62 species with SLA and 67 species with height mean values.

As for *Dataset II (Off-site data and taxonomic imputation)*, we used the 44 and 58 species for which no imputation was needed (i.e. with records available in TRY) to evaluate the accuracy of the phylogeny-based imputed values, by comparing trait means based on TRY records with those borrowed from their suggested phylogenetic equivalents.

#### Dataset IV—Off-site data and bhpmf imputation [*n* = 80]

Again, as for *Dataset II (Off-site data and taxonomic imputation)* and *Dataset III (Off-site data and phylogenetic imputation)*, we retrieved mean SLA and height for 44 and 58 species, respectively, from available records in TRY. In this dataset, imputation of missing values for the remaining species was made using R package *BHPMF* (Hierarchical Bayesian Probabilistic Matrix Factorization; Fazayeli et al. (2017)), which takes advantage of the correlations among matrices (Schrodt et al. 2015)—in this case trait and taxonomic hierarchy matrices—to impute the missing traits. To take full advantage of among-traits correlations, in addition to publicly available SLA and height records from TRY, we also used seed mass records to support the imputation. We were able to estimate mean SLA and height values for all species with missing information except two; there was no data for any of the three focal traits for these two species.

As with the previous two datasets, we used the 44 and 58 species for which no imputation was needed (i.e. with records available in TRY) to evaluate the accuracy of the imputation using probabilistic matrix factorization, by comparing trait means estimated through this method with those based on TRY records.

#### Dataset V—On- & off-site data [*n* = 82]

Bayesian methods provide a way to benefit from previous knowledge of trait global distributions (priors) to improve the (posterior) estimation of trait means from a small amount of records. We use a Bayesian model to update prior knowledge available from other sources (off-site records at global scale available in TRY) with on-site trait information, as presented in *Dataset I (On-site data)*:

TRAIT_t_ ~ Normal (mu_t,s_, sd_t_),

mu_t,s_ ~ Normal (prior_mu_µ_t,s_, prior_mu_σ_t,s_),

where TRAIT stands for each on-site observation of trait t, and was log-transformed and standardised, mu and sd are the posterior estimated mean and standard deviation of species s and trait t, and prior_mu_µ and prior_mu_σ are the prior mean and standard deviation of species s and trait t.

We estimated the prior distributions of SLA and height for the 82 species in *Dataset I (On-site data)* using the same modelling approach and collection of public records from TRY as for the imputation in *Dataset II (Off-site data and taxonomic imputation)*. However, this time we limited the global dataset to a maximum of 5 random records for each species (7,048 observations for SLA; 17,602 for height) to ensure equal contributions of priors and on-site records, which included on average 5 records per species.

#### Dataset VI—All off-site data [*n*_SLA_ = 73, *n*_height_ = 105]

This dataset included all species, out of the potential 157, with trait records available through TRY. Unlike the previous datasets, the number and identity of species included in this dataset was constrained by the amount of information already available (in this case, through the TRY database), rather than by other limitations such as the study design or field effort. An average of 9 records of SLA were available for 73 species and an average of 12 records of height for 105 species.

## Correlation across datasets

For both SLA and height, we calculated the correlation of species-level mean values between: (i) on-site and off-site records, using Pearson’s correlation coefficient (r), and (ii) pairs of datasets, using Spearman's correlation coefficient (rho, ρ). Since Spearman’s rho assesses monotonic relationships between two variables, whether they are linear or not, it reflects whether the rank order of species holds across datasets, even if their mean trait values differ.

## Case study: species invasiveness as a function of traits

We investigated the implications of different methodological choices required to build trait datasets on the correlation between plant traits and species’ invasive ability (i.e. invasiveness) of 157 exotic plants (Fig. 1). Invasiveness was measured in two ways: as the species’ maximum local abundance and as the species’ maximum spread rate in Victoria, Australia (Catford et al. 2016). Local abundance for each species was defined as their observed maximum relative cover across ~ 30,000 plots of remnant vegetation (Victorian Biodiversity Atlas). Spread rate was estimated as the maximum slope of a hierarchical sigmoid growth model based on data describing geographic spread over time, Palma et al. (2021a) combining records from the Victorian Biodiversity Atlas and the Australasian Virtual Herbarium. Records before 1900 were removed to prevent geospatial inaccuracy in the location data.

Plants’ ability to invade natural habitats has been previously linked to the leaf economic spectrum (Lake and Leishman 2004; Hamilton et al. 2005; Leishman et al. 2007; Gallagher and Leishman 2015; Gallagher et al. 2015; Buru et al. 2016) and plant growth rate (Radford and Cousens 2000; Bass et al. 2006; van Kleunen et al. 2010b; Buru et al. 2016). Recent work with exotic forbs and grasses in Victoria revealed that: (i) species’ local abundance was negatively correlated with SLA and positively correlated with seed mass; (ii) spread rate was positively correlated with height and negatively correlated with seed mass; and (iii) the probability of being classified as invasive increased with residence time (Palma et al. 2021a). Moreover, annual and perennial plants have been found to differ in their invasion dynamics, with annual species showing higher establishment than perennial counterparts (Palma et al. 2017; Catford et al. 2019).

We built twelve linear models; six of them with local abundance as the response, and six with spread rate as the response. Models for species’ local abundance included SLA, seed mass and minimum residence time as explanatory variables, whereas models for spread rate included plant height, seed mass and longevity. Species’ longevity was described as annual/biennial or perennial, and extracted from Richardson et al. (2011). Seed mass was collected from the Seed Information Database (Royal Botanic Gardens Kew 2020). Minimum residence time was calculated as the number of years since the first record of the species in Victoria as registered by the Australasian Virtual Herbarium (AVH 2020) after 1900. SLA and height mean values differed across the six models following the six datasets described in the previous section “Plant species and compilation of trait datasets”. Seed mass, longevity and minimum residence time data did not change across models.

We used one value to represent the seed mass of each species, i.e. we did not to evaluate seed mass in the same way that we evaluated SLA and height. We did this for two reasons. First, unlike height and SLA (Albert et al. 2010; Jung et al. 2010) there is evidence that seed attributes, such as seed mass, show little variability within species (Harper et al. 1970; Kazakou et al. 2014; Borgy et al. 2017), and, therefore, dissimilarities associated with methodological choices and their subsequent effects on ecological inference are expected to be minimal. Second, due to the large geographic distances between species’ populations (and our homes), we lacked the required resources to organise multiple trips to each site that coincided with availability of individual species’ seeds. All models took the form of:

Invasiveness ~ Normal (mu_s_, sd),

mu_s_ = α + Σ (β_t_ * TRAIT_s,t_),

where s represents each species, and β the effect of a given trait t on the invasion ability of plants when multiple traits are considered simultaneously. Local abundance and spread rate were logit- and log-transformed, respectively (Fig. S2) and then standardised; SLA, height and seed mass were log-transformed and standardised; minimum residence time was standardised; longevity was a binary variable with annual/biennial as the reference class and perennial as the alternative. For each model, we calculated the deviance explained (*R*^2^).

## Results

### Use of multiple sources and methods to build trait datasets

**Fig. 2:**
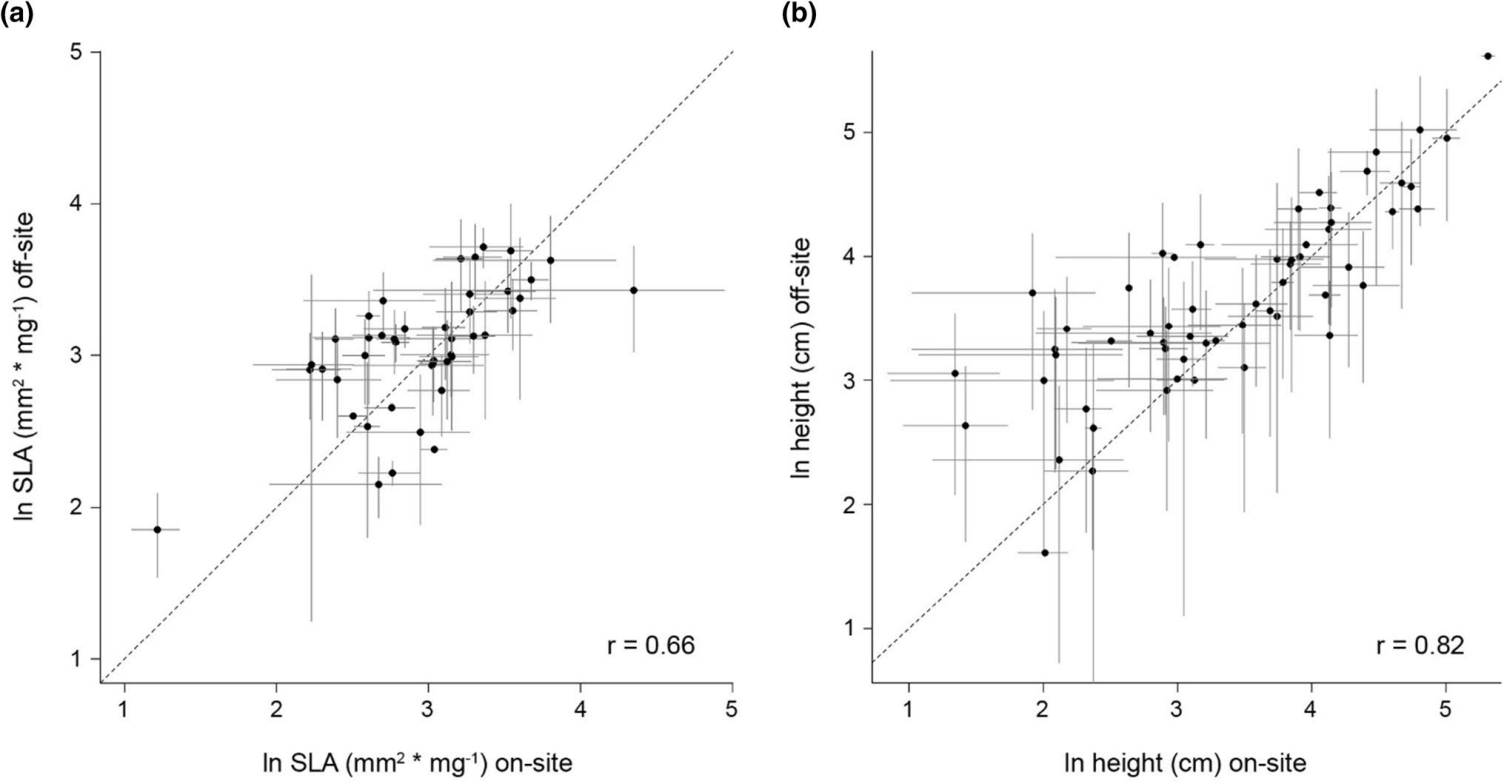
Correlation between natural logarithm of mean trait values calculated from on-site records (collected in Victoria) and off-site records (collected from TRY) for **a** SLA (*n* = 44) and **b** height (*n* = 58). Black dots and grey lines represent species-level means and standard deviations, respectively. Pearson’s *r* shown for each panel. The 1:1 relationship is represented by a dashed line

Out of the 82 species considered for *Datasets I to V*, 38 and 24 species lacked off-site records in TRY for SLA and height, respectively (Fig. 1). For the species with both on-site and off-site trait records (*n* = 44 for SLA; *n* = 58 for height), we found a high correlation between sources (i.e. collected in Victoria vs. collected through TRY; Pearson’s *r* ≥ 0.65, Fig. 2), reflecting that species’ rank order for both traits were relatively well conserved between sources (Fig. S3). The largest differences appeared at the lower end of the trait values in Victoria (e.g. shortest species as measured on-site in Victoria were taller in TRY).

**Fig. 3:**
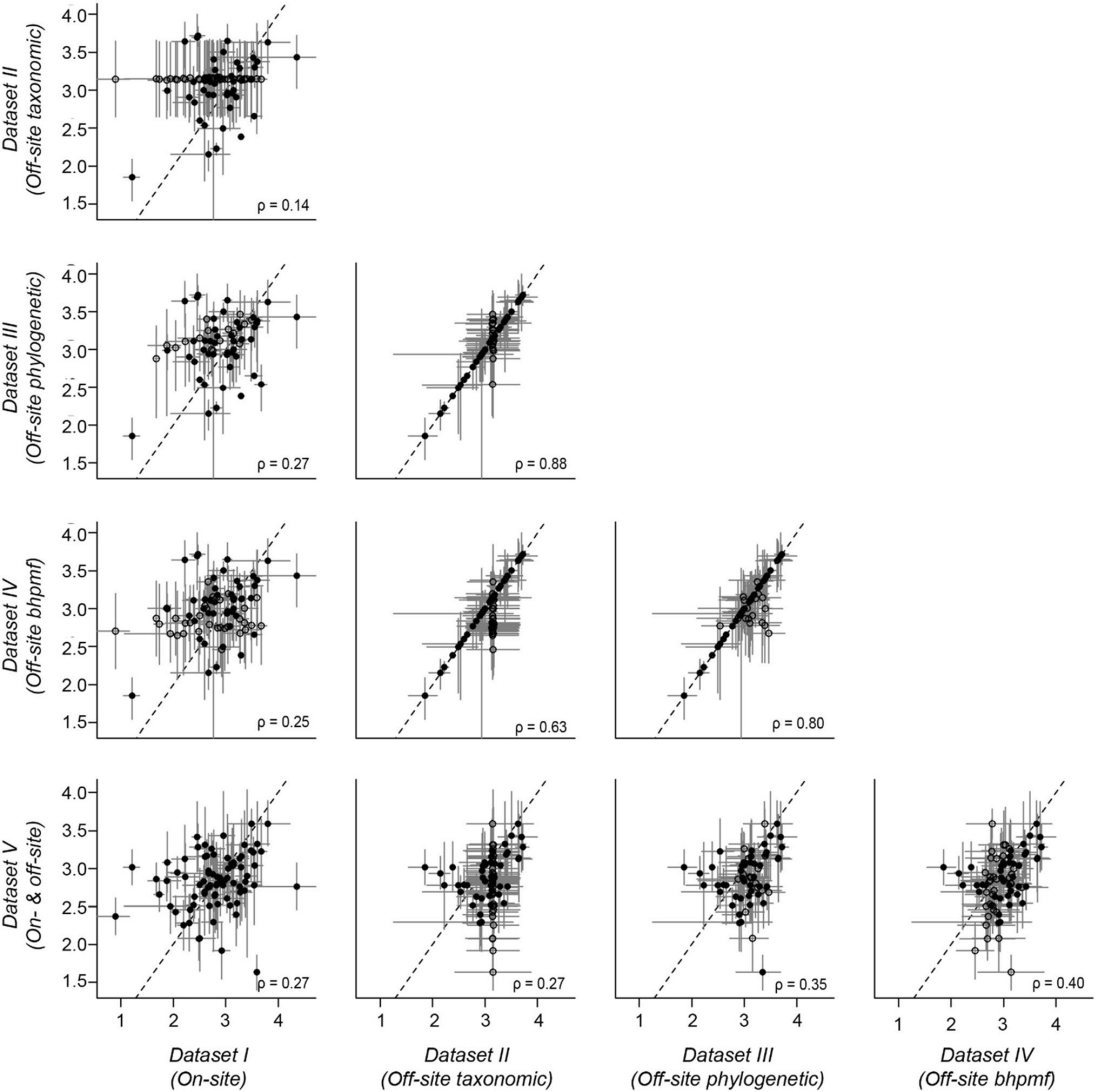
Correlation between natural logarithm of SLA values in pair-wise comparisons among *Datasets I* to *V* (see Methods). Black dots and grey lines represent species-level means and standard deviations (or 95% credible intervals), respectively. Filled dots represent species with measured mean vales and open dots represent species with imputed mean values. Spearman’s correlation rho, ρ, shown for each panel. Dataset *VI (All off-site data)* not shown because it includes a slightly different set of species (Fig. 1) and is thus not directly comparable

**Fig. 4:**
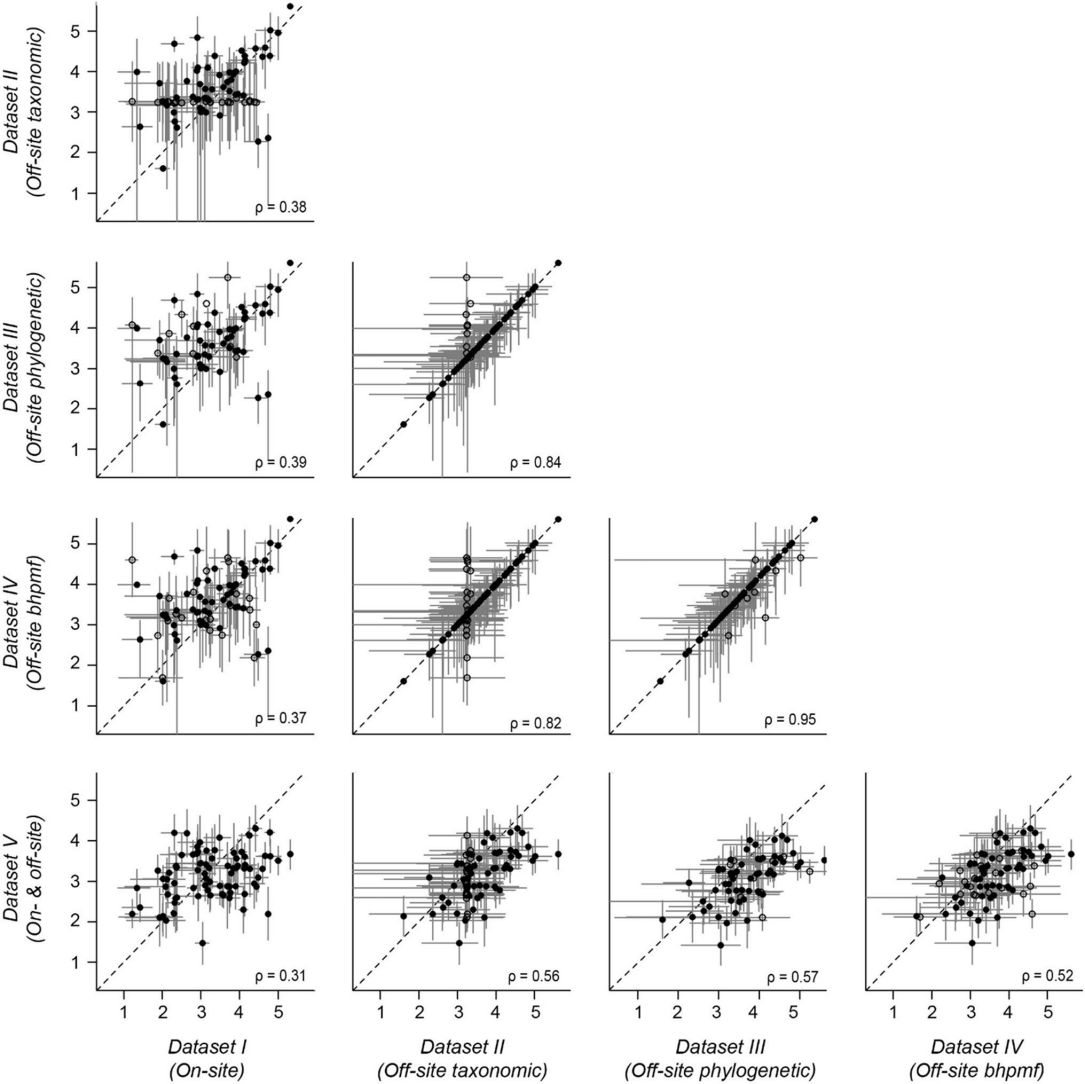
Correlation between natural logarithm of height values in pair-wise comparisons among *Datasets I* to *V* (see Methods). Black dots and grey lines represent species-level means and standard deviations (or 95% credible intervals), respectively. Filled dots represent species with measured mean vales and open dots represent species with imputed mean values. Spearman’s correlation rho, ρ, shown for each panel. Dataset *VI (All off-site data)* not shown because it includes a slightly different set of species (Fig. 1) and is thus not directly comparable

**Fig. 5:**
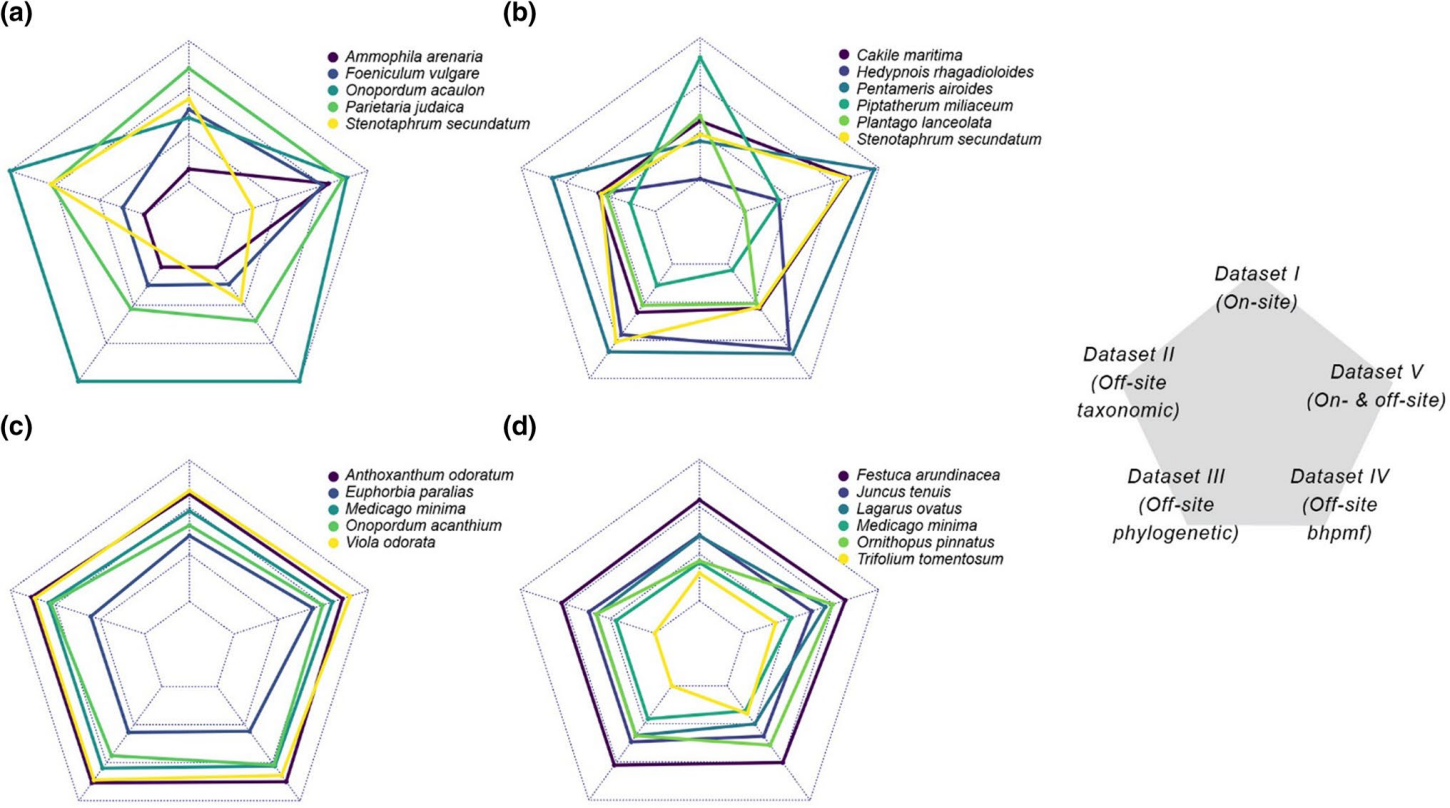
Examples of species with **a, b** highly variable or **c, d** relatively consistent mean **a, c** SLA and **b, d** height across *Datasets I* to *V*. Counterclockwise from upper tip, panels presents *Dataset I (On-site data)*, *Dataset II (Off-site data and taxonomical imputation), Dataset III (Off-site data and phylogenetic imputation)*, *Dataset IV (Off-site data and bhpmf imputation)* and *Dataset V (On- & off-site data)*. Dataset *VI (All off-site data)* not shown because it includes a slightly different set of species (Fig. 1) and is thus not directly comparable. Lines closer to the centre of each panel represent smaller trait values

**Fig. 6:**
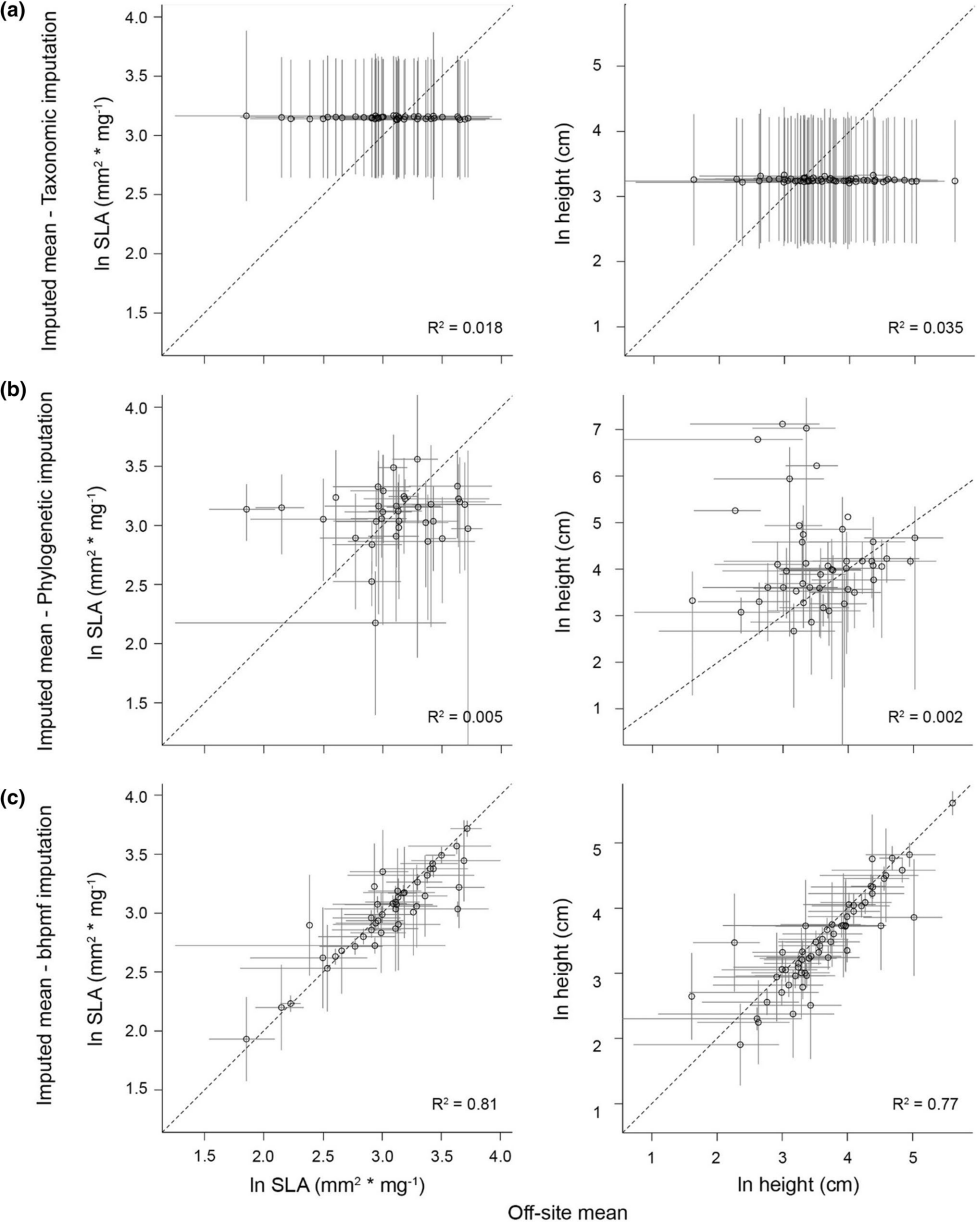
Species-level mean trait values (natural logarithm transformed) based on off-site records from TRY (*x*-axis) and mean trait values for the same species estimated through **a** taxonomic, **b** phylogenetic or **c** *bhpmf* imputation (*y*-axis). Open dots represent mean trait values and grey lines represent standard deviation. These comparisons were used to evaluate imputation methods, with higher *R*^2^ values reflecting better ability to retrieve available off-site values. See Appendix S4 for a discussion on the poor performance of the taxonomic imputation

The similarity among *Datasets I* to *V* was generally low (*ρ* < 0.57; Figs. 3,4), with mean trait values varying greatly across datasets for some species, while being relatively consistent for others (Fig. 5, see also Figs. S4–S6). An exception to this pattern was the relatively high similarity among *Dataset II (Off-site data and taxonomic imputation)*, *Dataset III (Off-site data and phylogenetic–imputation)* and *Dataset IV (Off-site data and bhpmf–imputation)*, all of which use off-site records and imputed missing values based on species’ relatedness (*ρ* = 0.63–0.95, Figs. 3, 4). These imputation methods yielded trait means that were highly uncertain and, in the case of the taxonomic imputation (*Dataset II*), centred around a small range of values on the centre of the trait distribution (Fig. S4), a feature also apparent during model evaluation (Fig. 6a). Although trait means estimated through phylogenetic imputation (*Dataset III*) covered a larger range of values than those estimated through taxonomic imputation (*Dataset II*), both methods showed low ability to accurately estimate off-site trait means (Fig. 6a,b; *R*^2^ < 0.05). Imputed traits with *bhpmf* (*Dataset IV*), on the contrary, were highly correlated with off-site mean trait values (Fig. 6c; *R*^2^ > 0.77).

## Case study: observed trait-invasion relationships based on different datasets

**Fig. 7:**
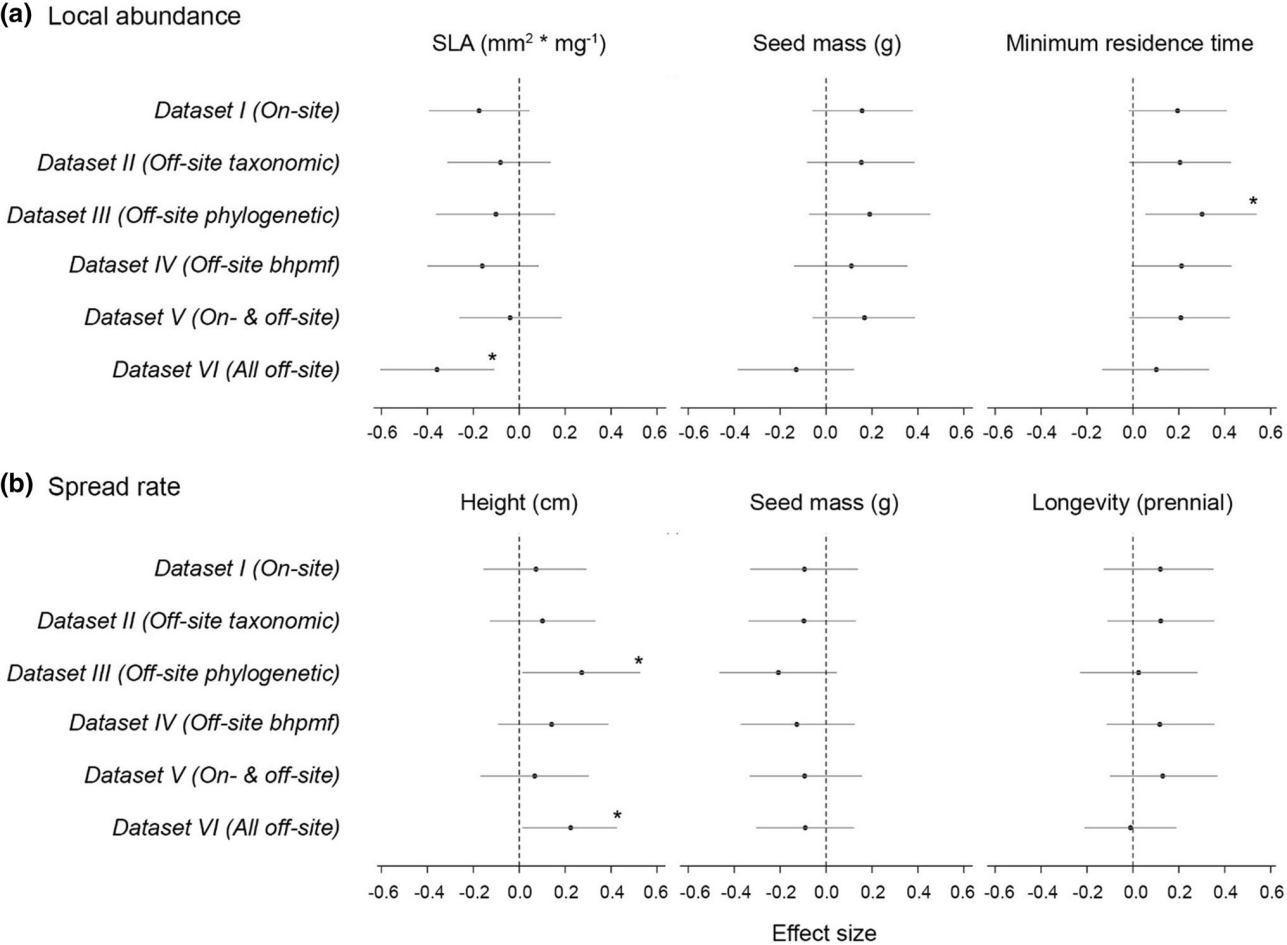
Effect of traits on exotic plants’ **a** local abundance and **b** spread rate. Dots and grey lines represent mean effects and 95% credible intervals, respectively. Consistent effects, with 95% credible intervals that do not overlap zero, are highlighted by an asterisk (see Methods). Unlike SLA and height, seed mass, minimum residence time and longevity values were held constant across *Datasets I* to *VI*. *R*^2^ values for local abundance models from *Datasets I* to *VI* are 0.111 (*n* = 82), 0.087 (*n* = 82), 0.162 (*n* = 62), 0.107 (*n* = 80), 0.082 (*n* = 82) and 0.118 (*n* = 73); *R*^2^ values for spread rate models from *Datasets I* to *VI* are 0.035 (*n* = 82), 0.040 (*n* = 82), 0.099 (*n* = 67), 0.045 (*n* = 80), 0.035 (*n* = 82) and 0.048 (*n* = 105)

The main effect of plant traits on exotic species’ local abundance and spread rate changed little across datasets (Fig. 7). Both SLA and height showed some inconsistencies across models, including significant and non-significant correlations with the invasiveness metrics. However, differences across models using different datasets were not significant (Fig. S7). The deviance explained for the models was low (*R*^2^ < 0.17), the multicollinearity among predictors was negligible (variance inflation factor < 1.15) and the model residual plots showed no patterns (Fig. S8).

Compared to the other datasets, results were slightly different for *Dataset VI (All off-site data)*, especially for models on local abundance (Fig. 7a). Despite its values being held constant across models (we did not evaluate changes across datasets for this trait), seed mass showed opposite relationships with local abundance for *Dataset VI* compared to *Datasets I* to *V*. However, when uncertainty was considered, none of the apparent differences in trait-invasion relationships across datasets were statistically significant (Fig. S7). We found similar patterns when a uniform sample size (*n* = 62 for local abundance, *n* = 67 for spread rate) was used across models (Fig. S9).

## Discussion

We investigated whether methodological choices made when building traits datasets affected: (1) rank order of species in those datasets and (2) inferences about relationships between plant traits and exotic species invasiveness, represented by abundance and spread rate (Fig. 1, Table 1). To do that, we built six SLA and height datasets following alternative methodological approaches that combined different sources of trait records, imputation techniques and species selection criteria. We found that even though the correlation between sources of trait records (i.e. on- and off-site data for same species) was high (Fig. 2), species’ rank order varied slightly across the six datasets (Fig. S4). This reflected the high variation found in some species’ traits across datasets (Fig. 5) and the low effectiveness of some imputation techniques (Fig. 6a,b). Variation in species trait means across the six datasets did not, however, translate into large differences in observed relationships between exotic plant species’ traits and their abundance and spread rate in Victoria, Australia (Fig. 7). The direction and statistical significance of trait-invasion relationships varied across datasets for height, SLA, seed mass and minimum residence time. However, differences among models were not statistically significant for either plant local abundance or spread rate (Fig. S7), and explanatory power of the competing models was similar (*R*^2^ ranged from 0.04 to 0.16).

## Differences among trait datasets and implications for invasiveness studies

Trait data collected across different spatial scales, as well as from different regions, can differ because of local adaptation, phenotypic plasticity and/or clinal trait variations (Table 1). As such, trait datasets built from locally or regionally sourced records may differ to those built from globally sourced records. In our study, we found that species’ mean trait values calculated from on-site records collected across Victoria and off-site records collected from the global TRY database were correlated (Fig. 2). Previous studies have also found relatively consistent species’ ranks and mean trait values across different sources of trait records, spatial scales or environmental conditions (Garnier et al. 2001; Roche et al. 2004; Mokany and Ash 2008; Kazakou et al. 2014; Violle et al. 2015b; Borgy et al. 2017; Mudrák et al. 2019; Kattge et al. 2020). For example, studies comparing trait means between species’ native and introduced areas have found that measurements from native areas may be a reasonable proxy for trait values in invaded areas (Thébaud and Simberloff 2001; Mason et al. 2008; Ordoñez 2014).

Despite the correlation between trait means from the on-site and off-site records (Fig. 2, *r*_SLA_ = 0.66 and *r*_height_ = 0.82), which indicates relatively consistent species ranks, we did find some differences in trait means (Fig. S3). We also found that intraspecific trait variability increased with geographic scale. Trait dissimilarity across on-site records increased with geographic distance between records (Appendix S1, Fig. S10), and off-site records showed, overall, larger trait dissimilarity than on-site records, particularly for height (Fig. S11). The observed decline in trait dissimilarity at smaller spatial scales may indicate that some species’ trait values become less varied in response to local ecological conditions in Victoria, compared to environmental conditions across species’ global distribution range. Such a trend would be consistent with effects of environmental filtering (Kraft et al. 2015; Pearson et al. 2018). The broader geographic extent (sensu Wiens (1989)) of the globally sourced off-site records would likely capture higher environmental heterogeneity than the on-site records from Victoria, and would thus have greater diversity of environmental filters and, accordingly, trait values.

Unlike the source of trait records, methodological choices aimed to increase sample size led to large inconsistencies among trait datasets in our study (Figs. 3,4). We increased sample size by either: i) imputing missing off-site trait values (*Datasets II–IV*); ii) evaluating all species for which off-site trait records were available, rather than only the species of interest for the ecological question at hand (*Dataset VI*); or iii) combining on-site and off-site records through Bayesian update (*Dataset V*). Like the simulation study of Johnson et al. (2020), we found that choice of imputation technique can lead to significant differences in trait mean values for some species (Fig. 5). However, in contrast to Johnson et al. (2020), we did not find that the low correlation among datasets built through different imputation methods (Figs. 3,4) translated into inconsistencies in trait-invasion correlations (Fig. 7).

Studies aiming to understand effects of methodological decisions, such as imputation of missing values, on the trait values of the resulting datasets are relatively new (Penone et al. 2014; Johnson et al. 2020). Our results confirm that the most reliable imputation techniques for missing trait values involve methods that use both phylogenetic relationships and variance–covariance matrices, such as *bhpmf* (*Dataset VI*) or Phylopars (not evaluated in this work; Penone et al. (2014)). More simplistic imputation techniques, in particular the taxonomic imputation used to build *Dataset II*, lead to poor representation of species’ mean trait values (see Fig. 6a). We suspect the reason behind its poor performance is related to the unequal availability of trait data in global databases across species, and their taxonomical and geographic biases (Violle et al. 2015a). For example, widespread species, which are also introduced to new regions more often (Blackburn et al. 2015), are better represented in trait databases, both in terms of number of species and in number of records. Taxonomy-based imputation borrows information predominantly from clades that are better represented, potentially amplifying existing biases in global trait databases, e.g. towards common species or geographic areas, and conveying them into imputed trait estimates (see Appendix S4).

## Recommendations for trait-based invasiveness studies

Like most, if not all, ecological studies, we were unable to determine the ‘true’ species’ mean SLA and height in our study. A lack of perfect information necessarily limits our ability to evaluate the fit of our six datasets and recommend which method(s) is better (e.g. which explains the most variance). Despite this limitation, our case study illustrates the consistency of results from six datasets that were built in different ways. In doing so, it exemplifies two common (conscious or unconscious) considerations made when trait datasets are built: i) at which scale(s) is the ecological process being studied? and ii) is there enough trait information to answer the ecological question?

In our case study, we wanted to investigate the correlation between species’ traits and two aspects of their invasiveness in Victoria, Australia—their spread rate and local abundance. While species introductions may be governed by drivers at larger scales (Pyšek et al. 2020), the performance of introduced species in the local environment is likely to be highly dependent on species’ functional traits. It is thus conceivable that the optimal way to understand the importance of plant traits on species’ spread and local abundance in Victoria is by building a dataset consisting of on-site records, i.e. *Dataset I* (Table 1)—but see discussion paragraph below (Pyšek et al. 2009; Martín-Forés et al. 2018; Hejda et al. 2019). *Dataset I (On-site data)* is the best representation of the species’ traits across the geographic area where the ecological patterns were evaluated in this study (Fig. 1). On-site collection of trait records accounts for biases associated with human preferences for introduction (Palma et al. 2021b), as well as local processes of adaptation. However, species’ trait values can be similar across native and introduced ranges (Thébaud and Simberloff 2001; Mason et al. 2008; Ordoñez 2014), which suggests that on-site data are not essential. Further, acquiring on-site data for exotic plants may not be possible for logistic reasons or because of the patchy and dynamic nature of exotic species’ distributions (idiosyncratic populations, low local abundance, known populations that have been managed, and unknown emerging populations), which may result in incomplete data collection (Pérez-Harguindeguy et al. 2013) (Fig S12).

If collecting a sufficient number of local records is unfeasible, or there is already a reasonable representation of the species of interest in available trait databases, we suggest that off-site records can be used as a proxy for exotic species’ traits in the study area (Table 1). Use of off-site records should still increase understanding of the links between plant traits and species invasiveness, as other studies have shown (Pyšek et al. 2009; Martín-Forés et al. 2018; Hejda et al. 2019). This approach could be particularly suitable to explore those dimensions of the invasion process related to species’ arrival or naturalisation. Use of off-site records can also enable comparison of trait values from a species’ native range and trait values from their introduced range (which may extend well beyond the focal study area). While such an examination was beyond the scope of our study, other studies have found that differences in trait means between native and introduced populations can help explain species invasiveness in the introduced range (Pyšek et al. 2009; Hejda et al. 2019). A large difference in trait values between native and introduced populations may, for example, indicate high phenotypic plasticity or rapid post-introduction evolution in introduced populations—attributes that are likely to facilitate invasion (Martín-Forés et al. 2017, 2018). Depending on the question of interest, it may thus be better to avoid averaging species trait values across their entire range, as we did here (*Datasets II* to *VI*), but instead consider splitting them into native and introduced ranges. In addition to considering whether it is best to use on-site or off-site records, when working with exotic species, we recommend that people consider whether it is best to use trait data from species’ native range only, species’ introduced range only, species’ entire range (i.e. native + introduced) or a comparison of the two.

When imputation is required to increase sample sizes (i.e. number of species studied), we recommend employing methods—such as *bhpmf* or Phylopars (Penone et al. 2014)—that use both phylogenetic relationships and trait correlations to estimate trait values, and avoiding methods that only rely on taxonomic or phylogenetic relationships. Combining on-site and off-site records in a statistically structured way (*Dataset V*) may represent a suitable alternative to formal imputation techniques (*Datasets II, III, IV*). Although we decided to give equal weight to on-site and off-site data, the influence of off-site records can be downweighted to stress the relative importance of the invading phenotypes (on-site records). Increasing sample size by Bayesian methods (*Dataset V*) has been largely unexplored so far, though, and further work is needed to determine the best way to estimate Bayesian priors to answer these invasion ecology questions.

## Conclusion

In this study, we use a case study of invasion to demonstrate the consistency of results from six datasets that were built in different ways. Even though we did not find that methodological choices for data compilation had large effects on ecological inference in our case study, we recommend: (1) using on-site records to understand locally or regionally based invasion processes (e.g. species’ local abundance and spread) whenever possible; and (2) transparency when reporting methodological decisions related to selection of study species and estimation of missing trait values.

## Supplementary Information

Below is the link to the electronic supplementary material.

Supplementary file1 (DOCX 44 kb)

Supplementary file2 (DOCX 14002 kb)

Supplementary file3 (DOCX 51 kb)

## Data Availability

Off-site trait records used in this manuscript are publicly available in the TRY database (https://www.try-db.org). On-site trait records collected in Victoria by the authors are publicly available at Zenodo: https://doi.org/10.5281/zenodo.4314008.

## Code availability

Code developed by the authors can be found in the Electronic Supplementary Material—Appendix S2.
