# Supplementary material for: Building trait datasets: effect of methodological choice on a study of invasion
Source: Oecologia. 2022 Aug 17;199(4):919–35. doi: 10.1007/s00442-022-05230-8 (PMC9464113; doi:10.1007/s00442-022-05230-8)
Supplement: Supplementary file 1 — Supplementary file1 (DOCX 44 kb) [file 442_2022_5230_MOESM1_ESM.docx]

**SUPPLEMENTARY MATERIAL – Figures S1 to S14**

**Palma et al. (2022) “Building trait datasets: effect of methodological choice on a study of invasion.” Oecologia**

**Figure S1.** Map of sampling locations in Victoria. Refer to Table S1 for location names and species’ sample sizes. Red dot represents Melbourne.


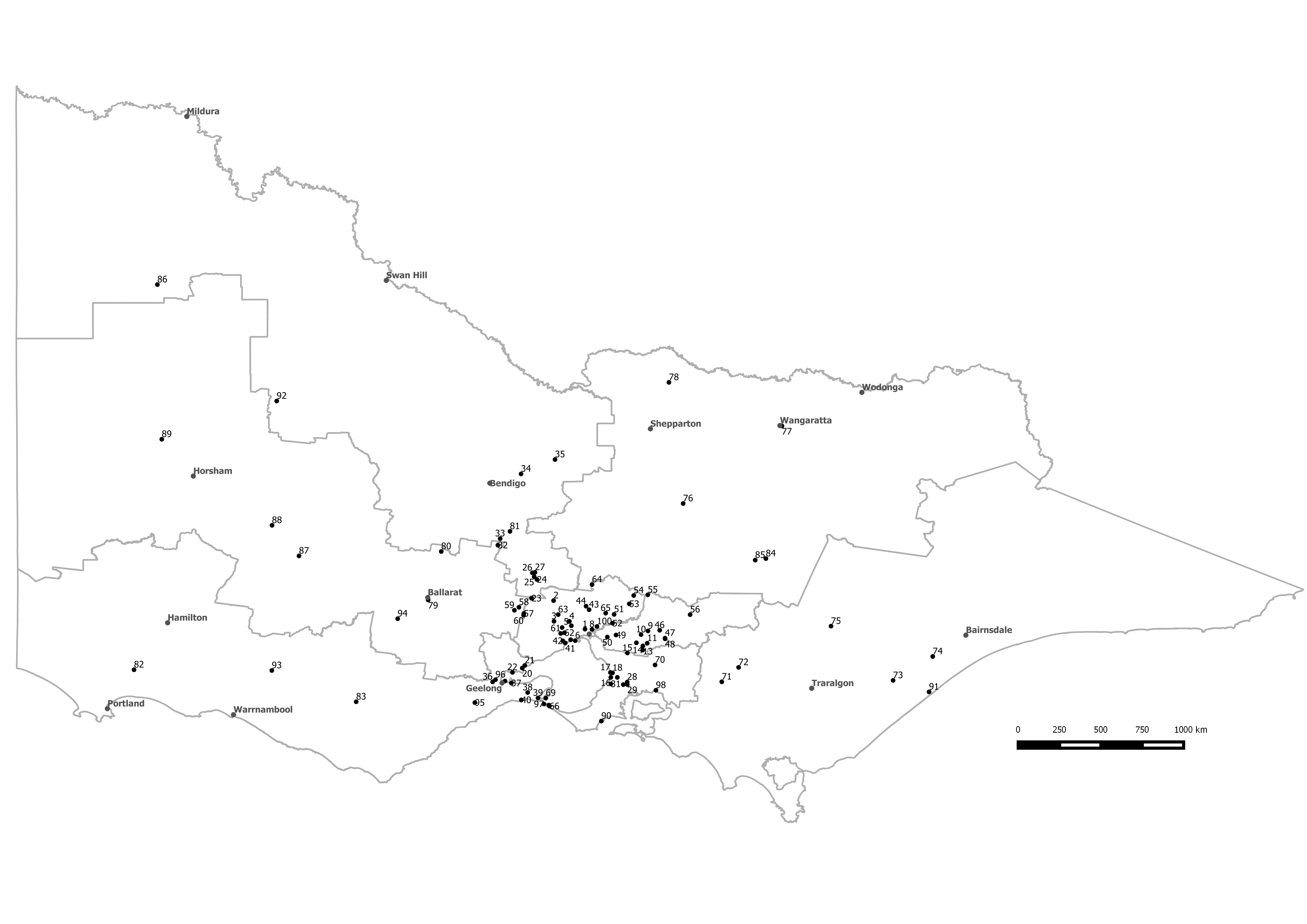

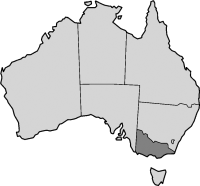


**
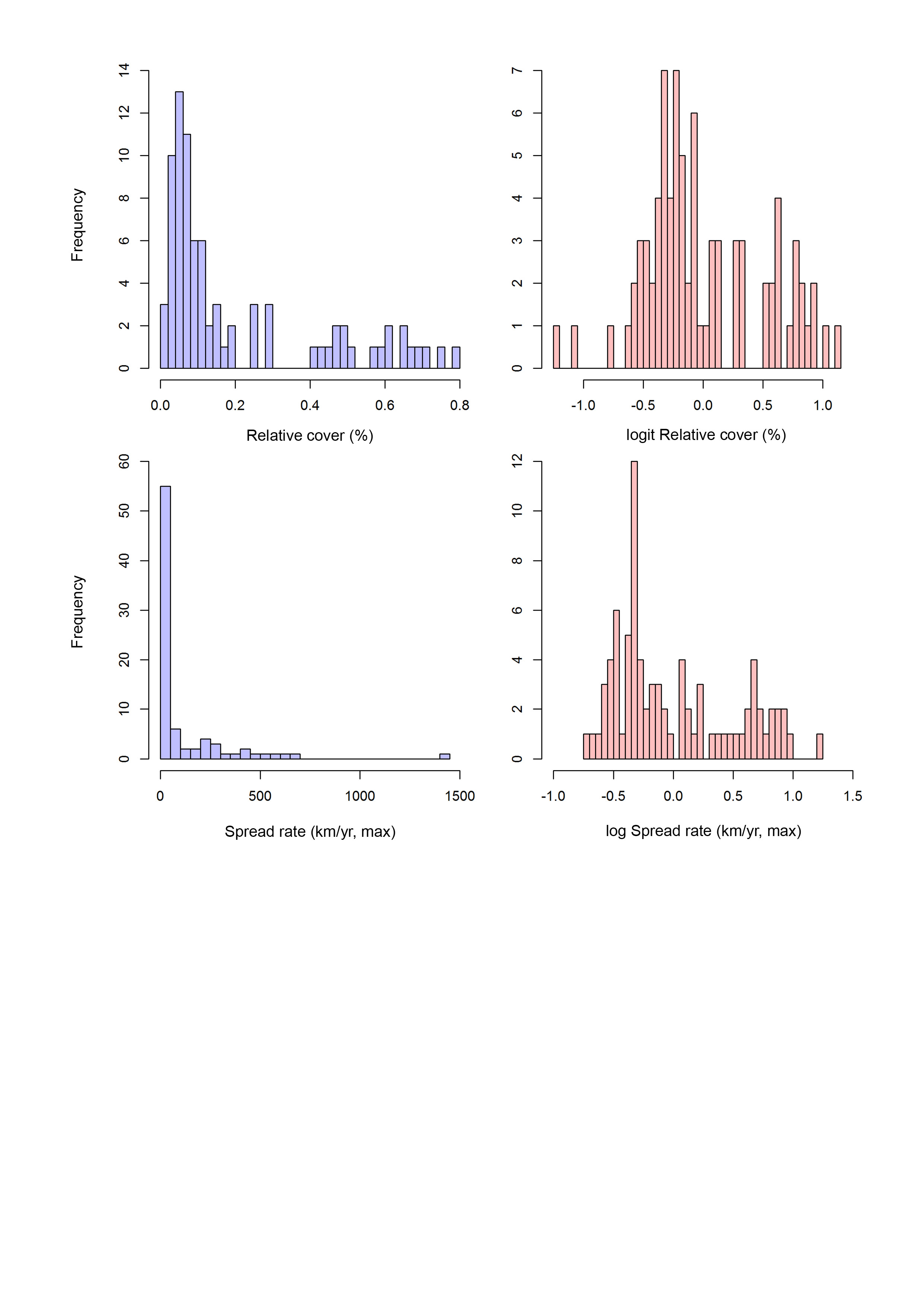
**

**Figure S2.** Distribution of values of the invasiveness metrics used as response in the case study analyses, without being transformed (left) and after being transformed (right).

**
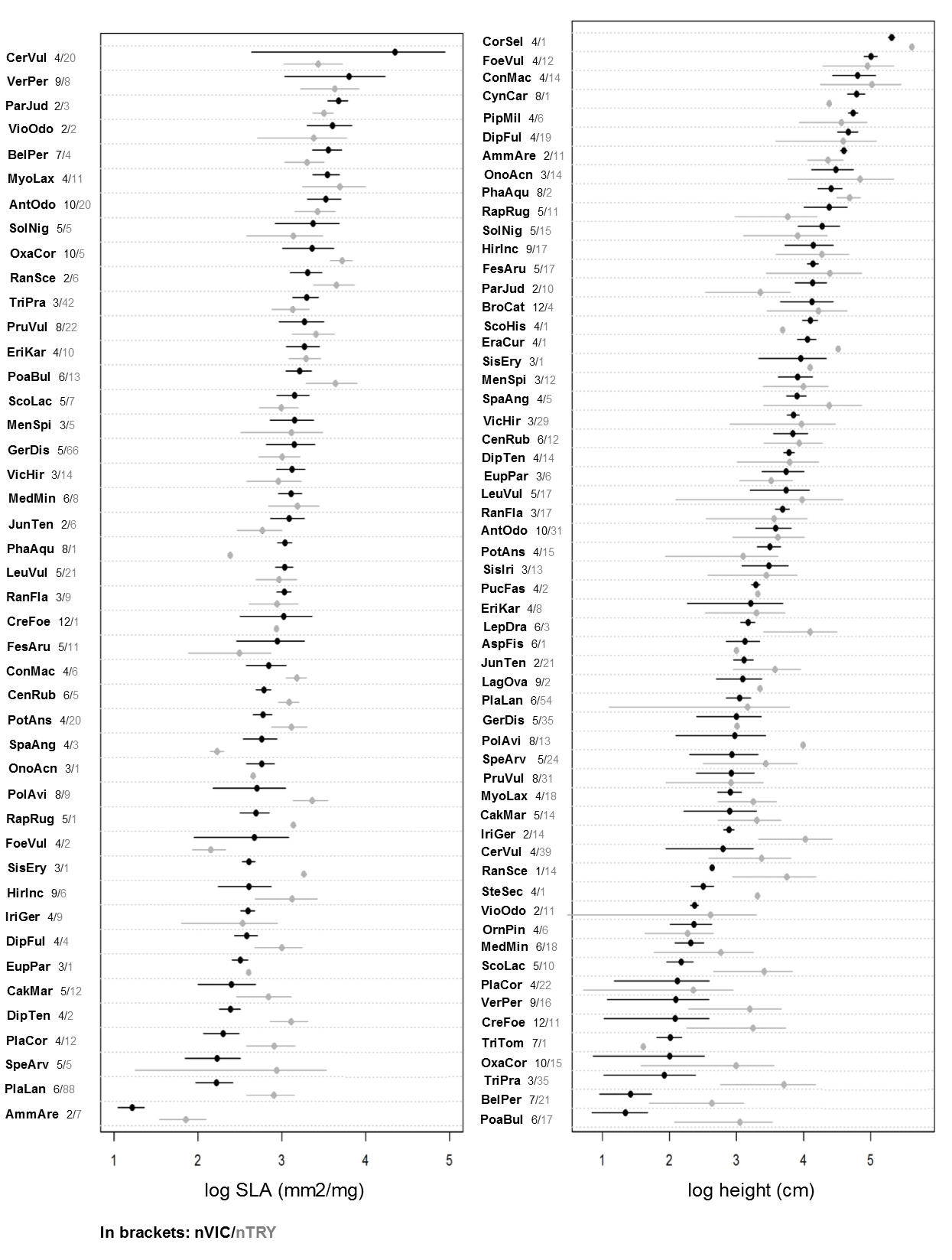
**

**Figure S3.** Rank of species following mean SLA (left panel) and height (right panel) found on-site (in black). In grey, species means calculated from off-site records. Dots and lines represent species’ means and standard deviations, respectively. Only species with both on-site and off-site records are shown (n_SLA_=44, n_height_=58), with the number of records from each source next to the species name – full species names provided in Table S2.

**
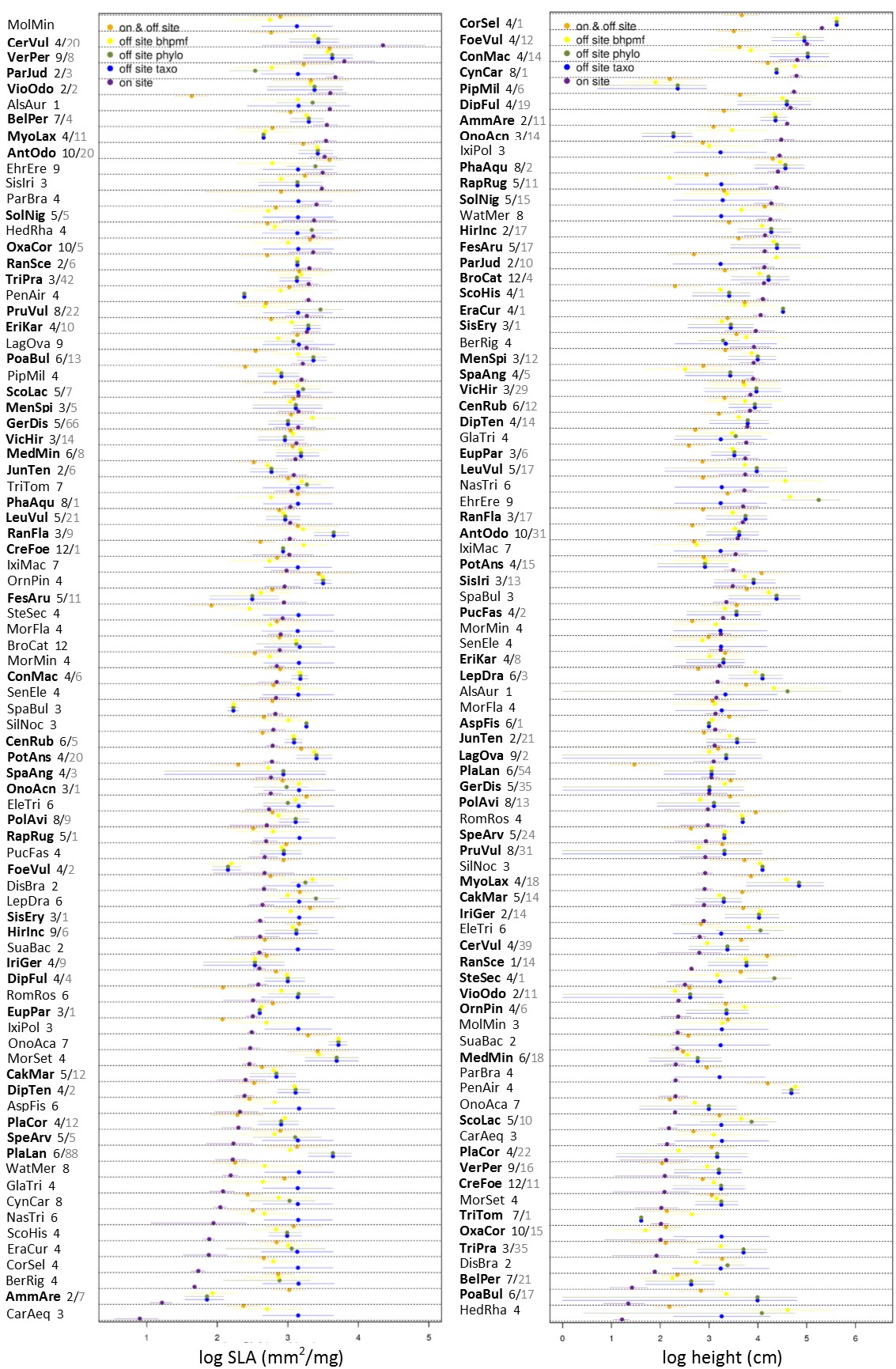
Figure S4.** Rank of species following mean SLA (left panel) and height (right panel) found in Victoria (*Dataset I – On-site data*, in purple). In blue, green and yellow, mean values of *Dataset* *II (Off-site data and taxonomic imputation*), *Dataset* *III (Off-site data and phylogenetic imputation*) and *Dataset* *IV (Off-site data and bhpmf imputation*). In orange, mean values of *Dataset* *V (On- & off-site data)*. Dots and lines represent species’ means and standard deviations, respectively. Species in bold had records in TRY. Full species names provided in Table S2.

**
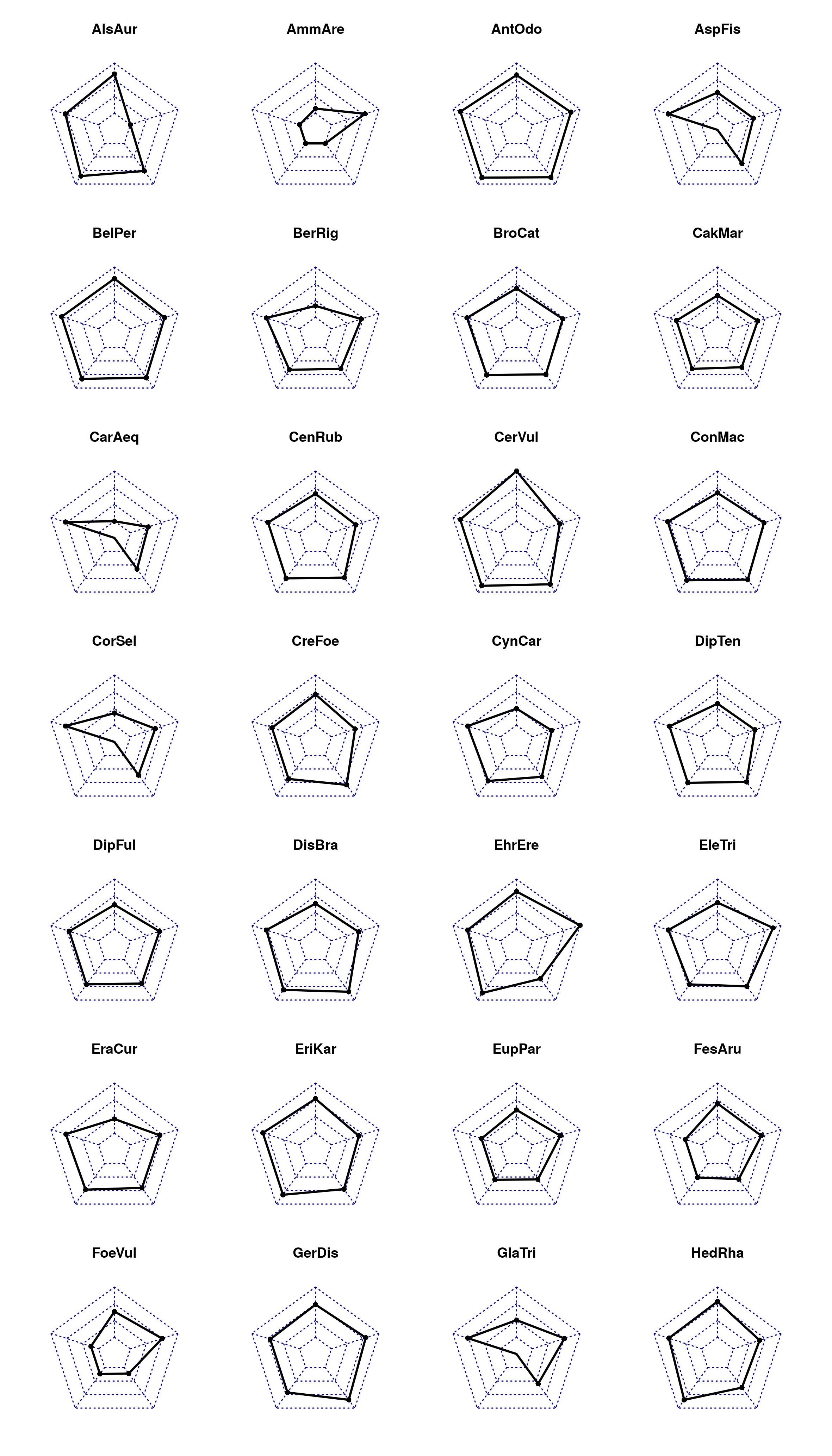
Figure S5**. Spider plots showing mean SLA for species in *Datasets I* to *V*. As in Figure 5, tips (from top anticlockwise) represent *Dataset I (On-site data)*, *Dataset* *II (Off-site data and taxonomical imputation)*, *Dataset* *III (Off-site data and phylogenetic imputation*), *Dataset* *IV (Off-site data and bhpmf imputation*) and *Dataset* *V (On- & off-site data).* *Dataset* *VI (All off-site data)* not shown because it does not cover the exact set of species, and the shared ones have the same values as *Datasets* *II, III* and *IV.* Lines closer to the centre of each panel represent smaller SLA values. Refer to Table S2 for full species names.


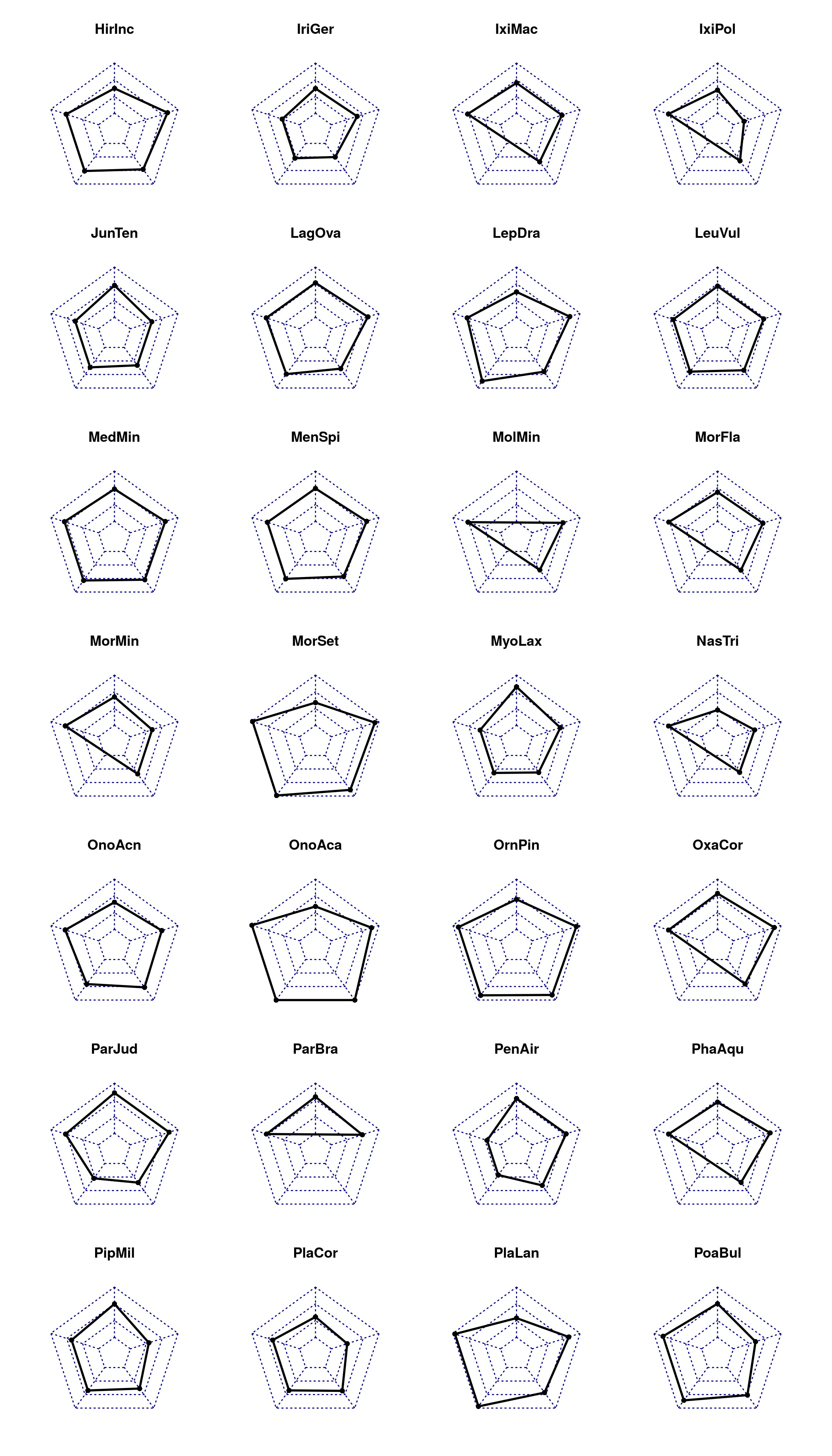
**Figure S5 Continued.**


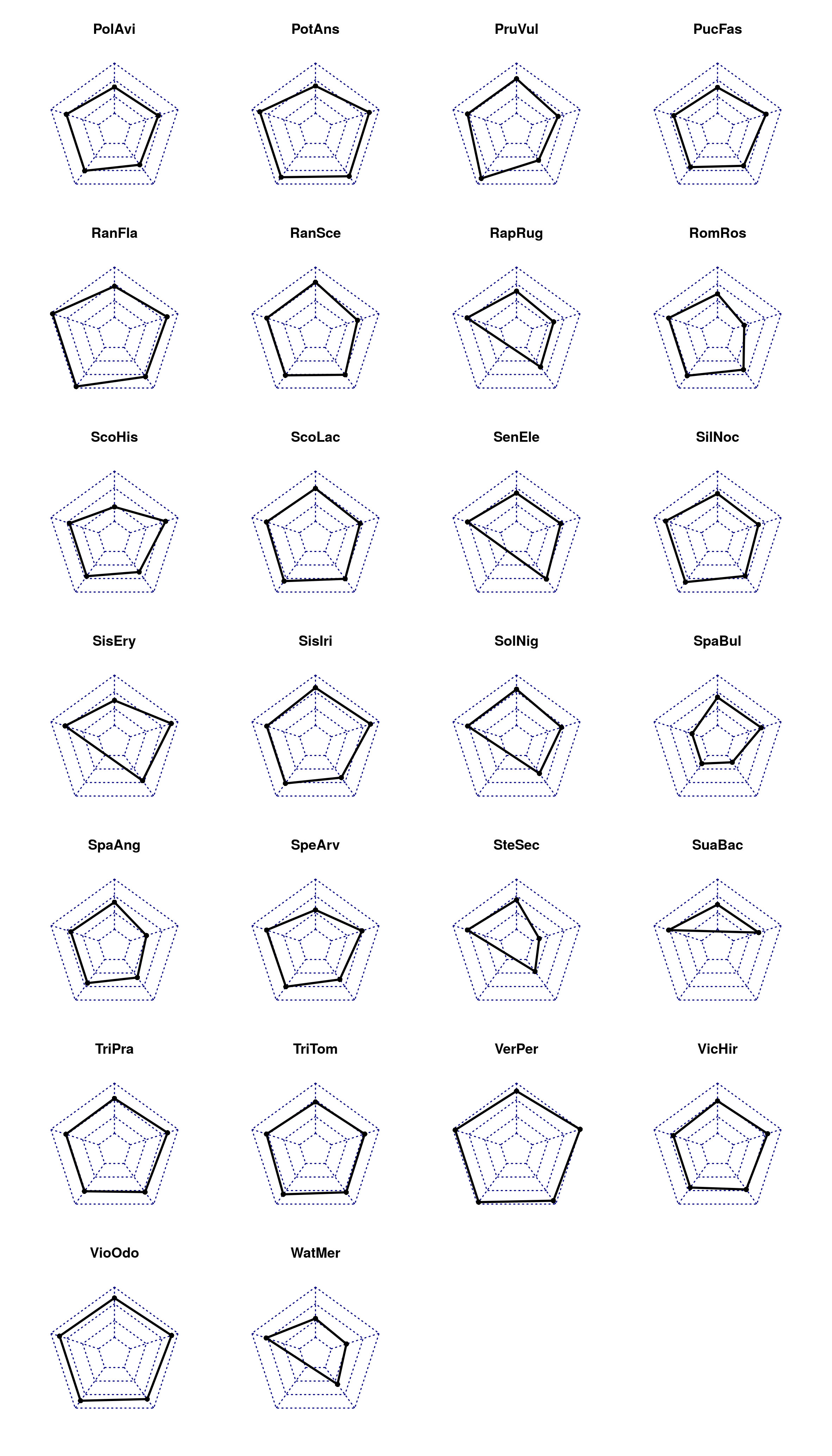
**Figure S5 Continued.**


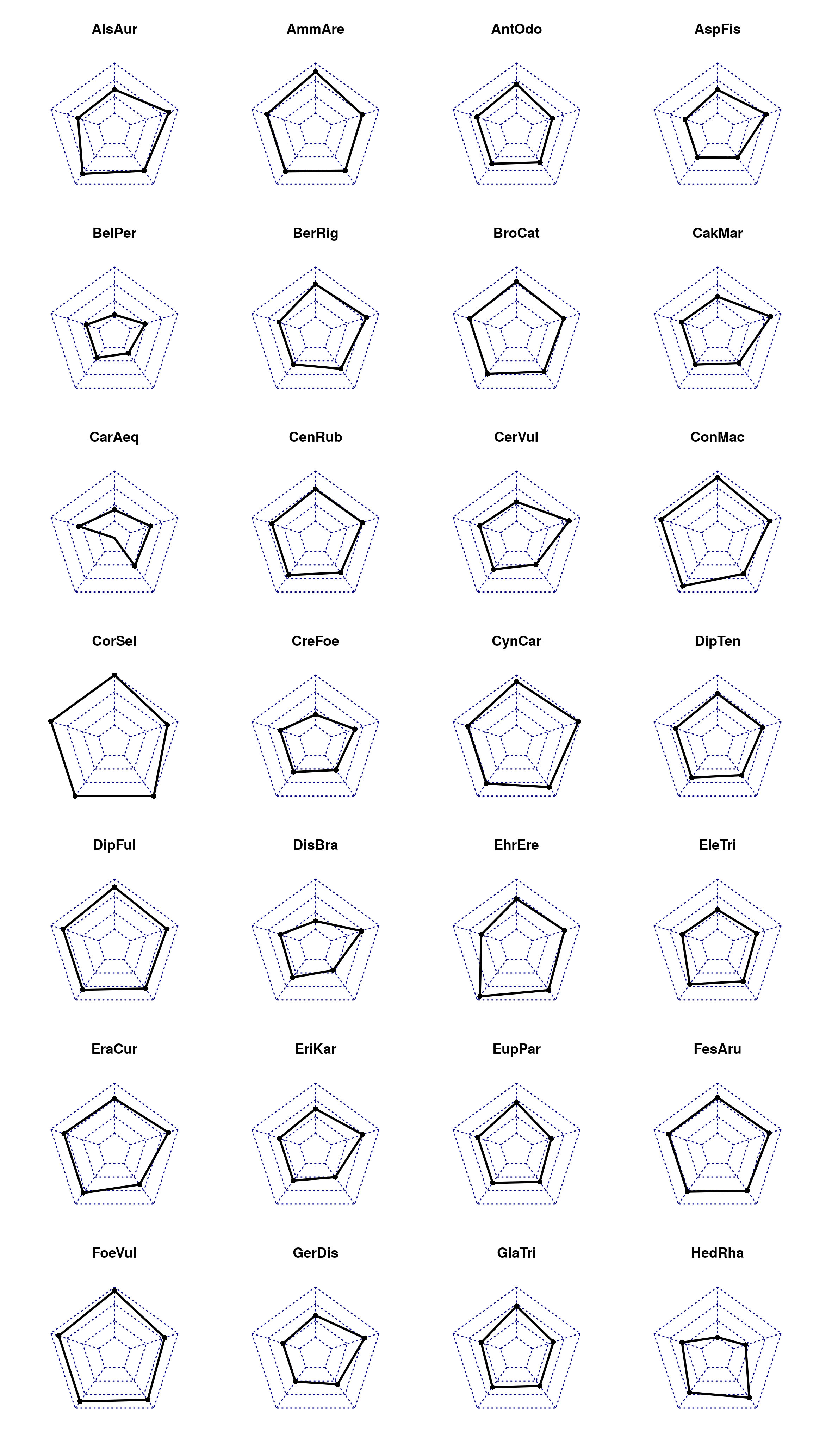
**Figure S6**. Spider plots showing mean height for species in *Datasets I* to *V*. As in Figure 5, tips (from top anticlockwise) represent *Dataset I (On-site data)*, *Dataset* *II (Off-site data and taxonomical imputation)*, *Dataset* *III (Off-site data and phylogenetic imputation*), *Dataset* *IV (Off-site data and bhpmf imputation*) and *Dataset* *V (On- & off-site data).* *Dataset* *VI (All off-site data)* not shown because it does not cover the exact set of species, and the shared ones have the same values as *Datasets* *II, III* and *IV.* Lines closer to the centre of each panel represent smaller height values. Refer to Table S2 for full species names.


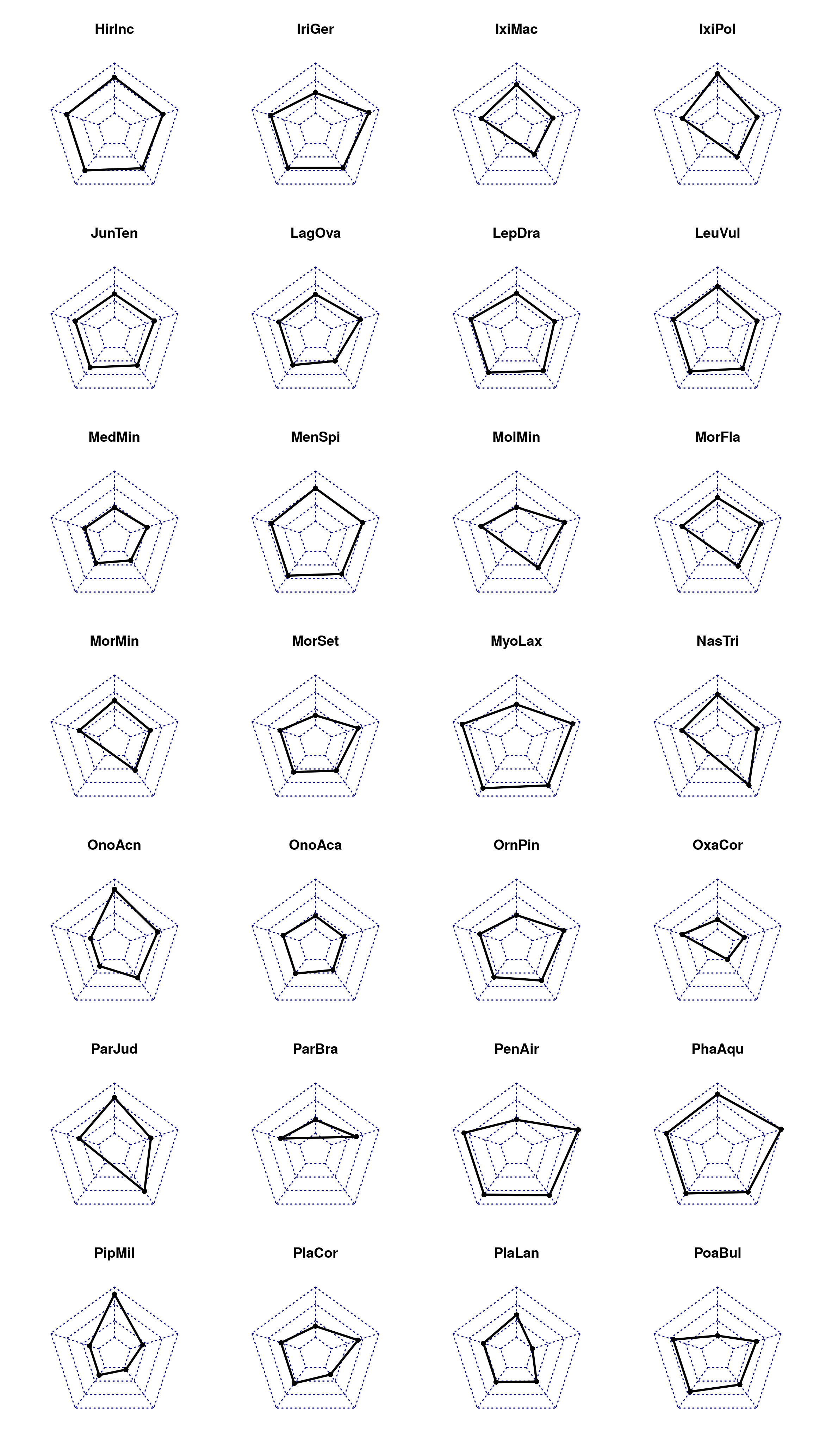
**Figure S6 Continued.**


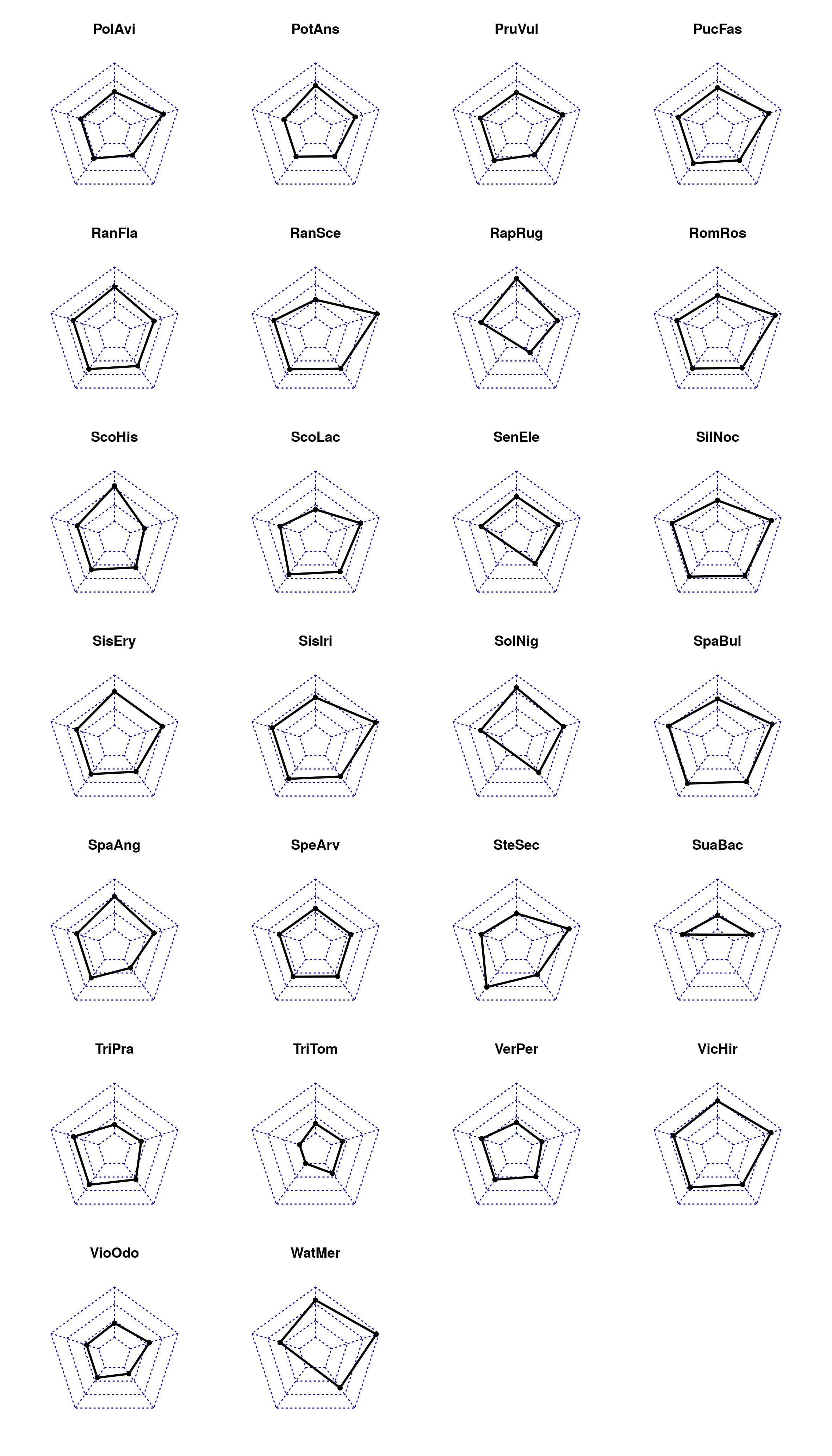
**Figure S6 Continued.**


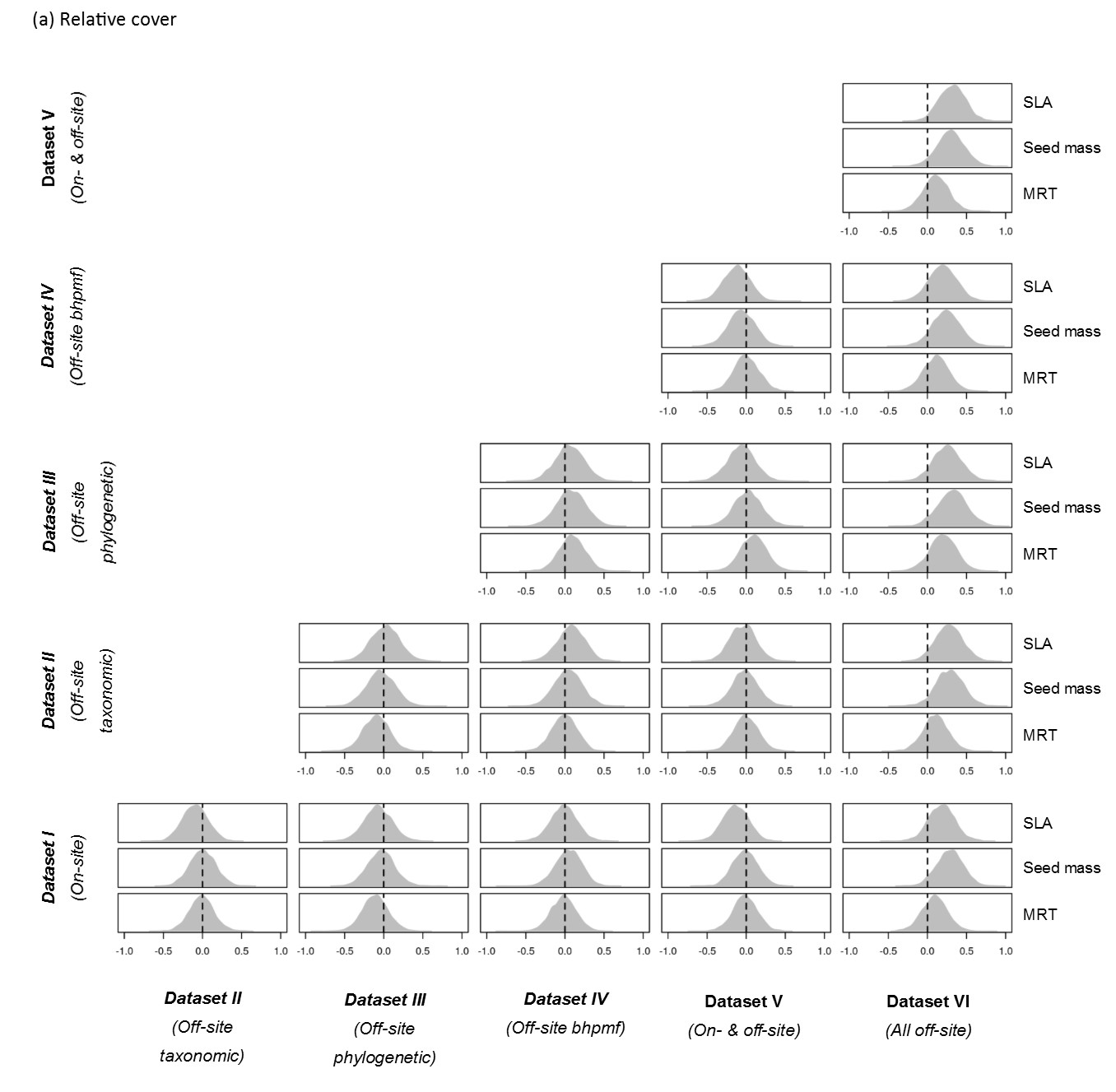


**Figure S7**. Formal comparison of the effect of traits on exotic plants’ local abundance (a) and spread rate (b) estimated by models across the six datasets. The vertical dashed line means no difference between trait effects estimated by two models. Right area from the line represents the effect calculated using the dataset on the y-axis is larger than the effect estimated using the dataset on the x-axis; left area represents the opposite.


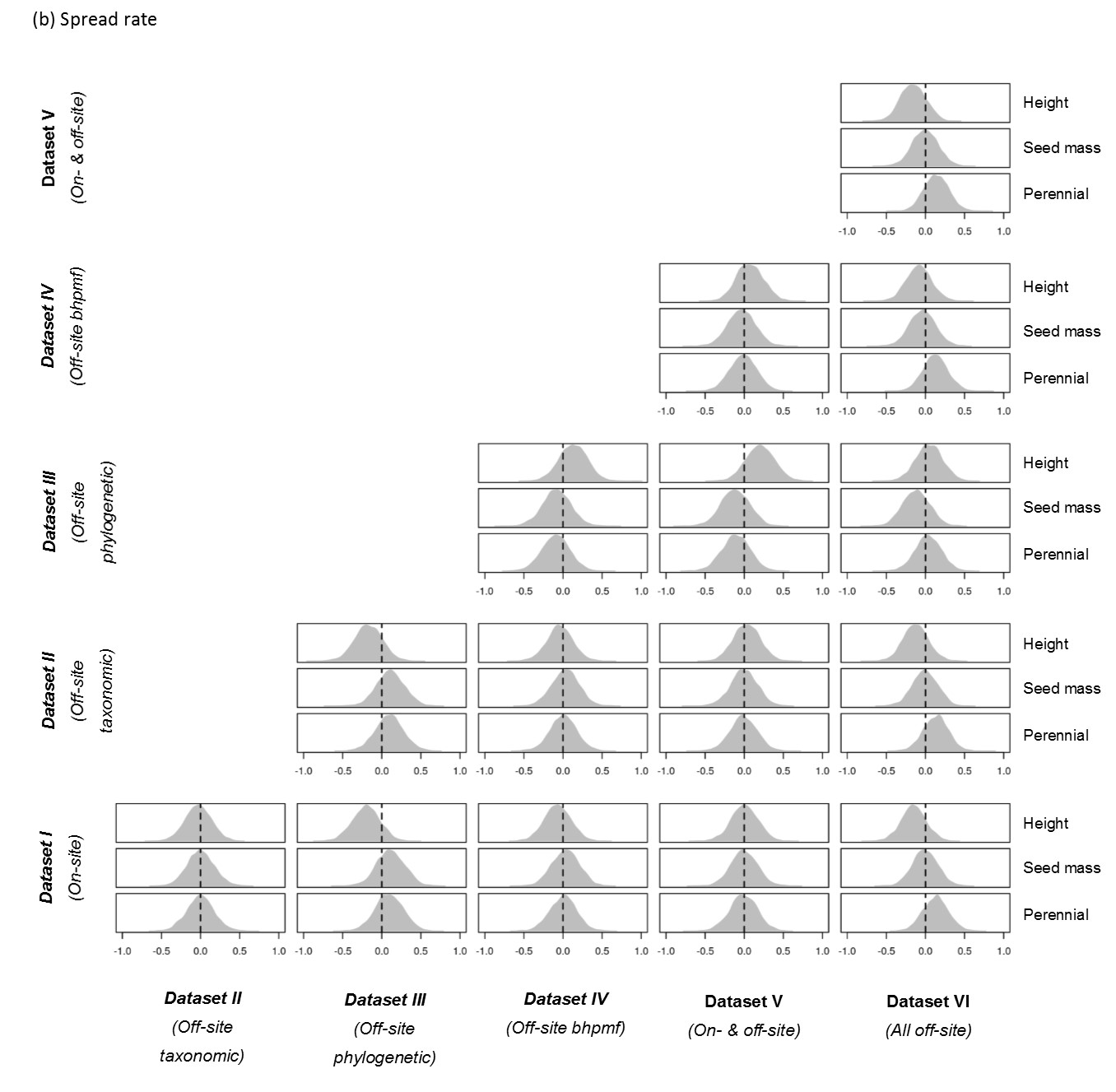


**Figure S7 Continued.**

**
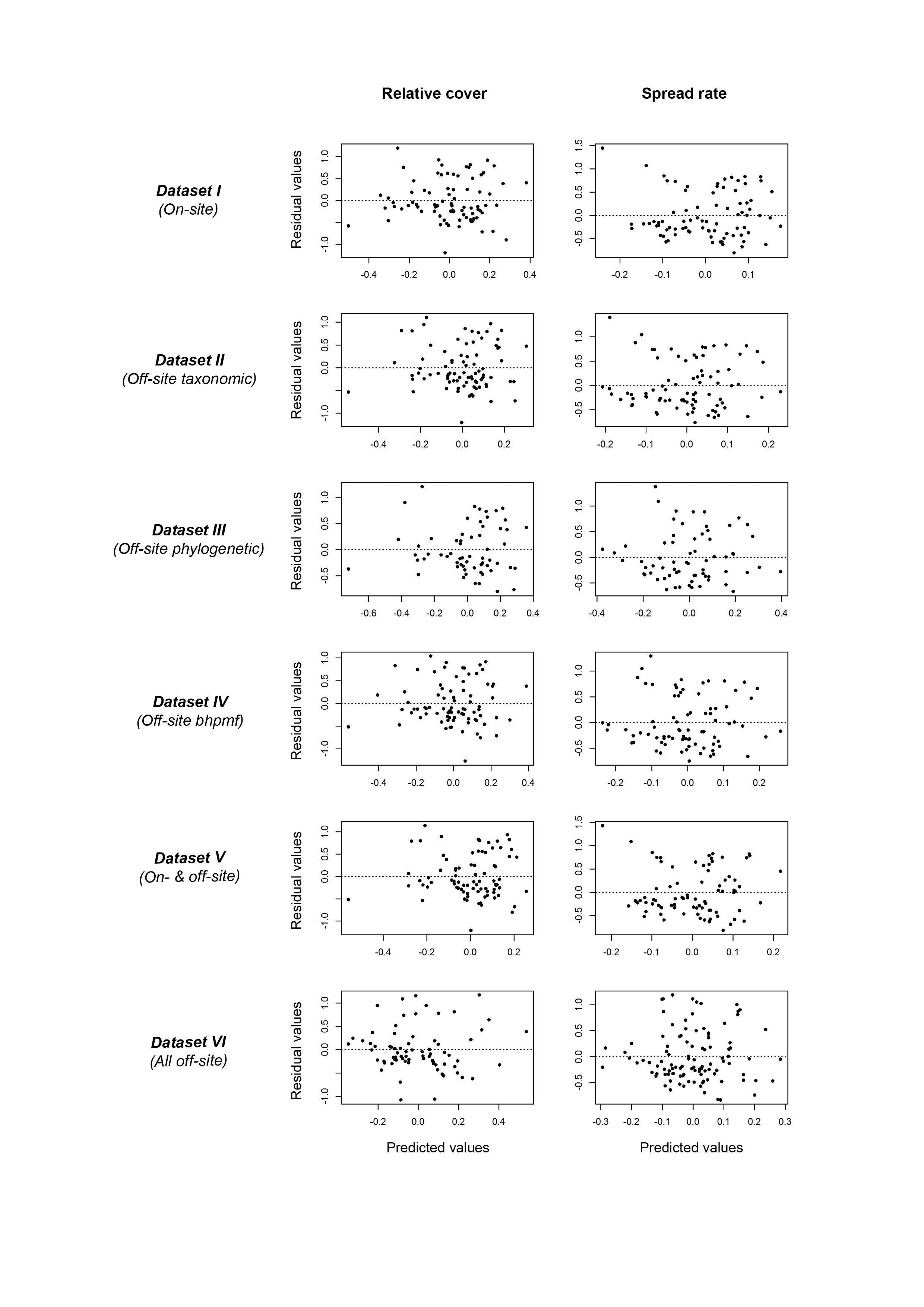
**

**Figure S8.** Residual plots of the invasiveness models of the case study - exotic plants’ local abundance on the left and spread rate on the right.


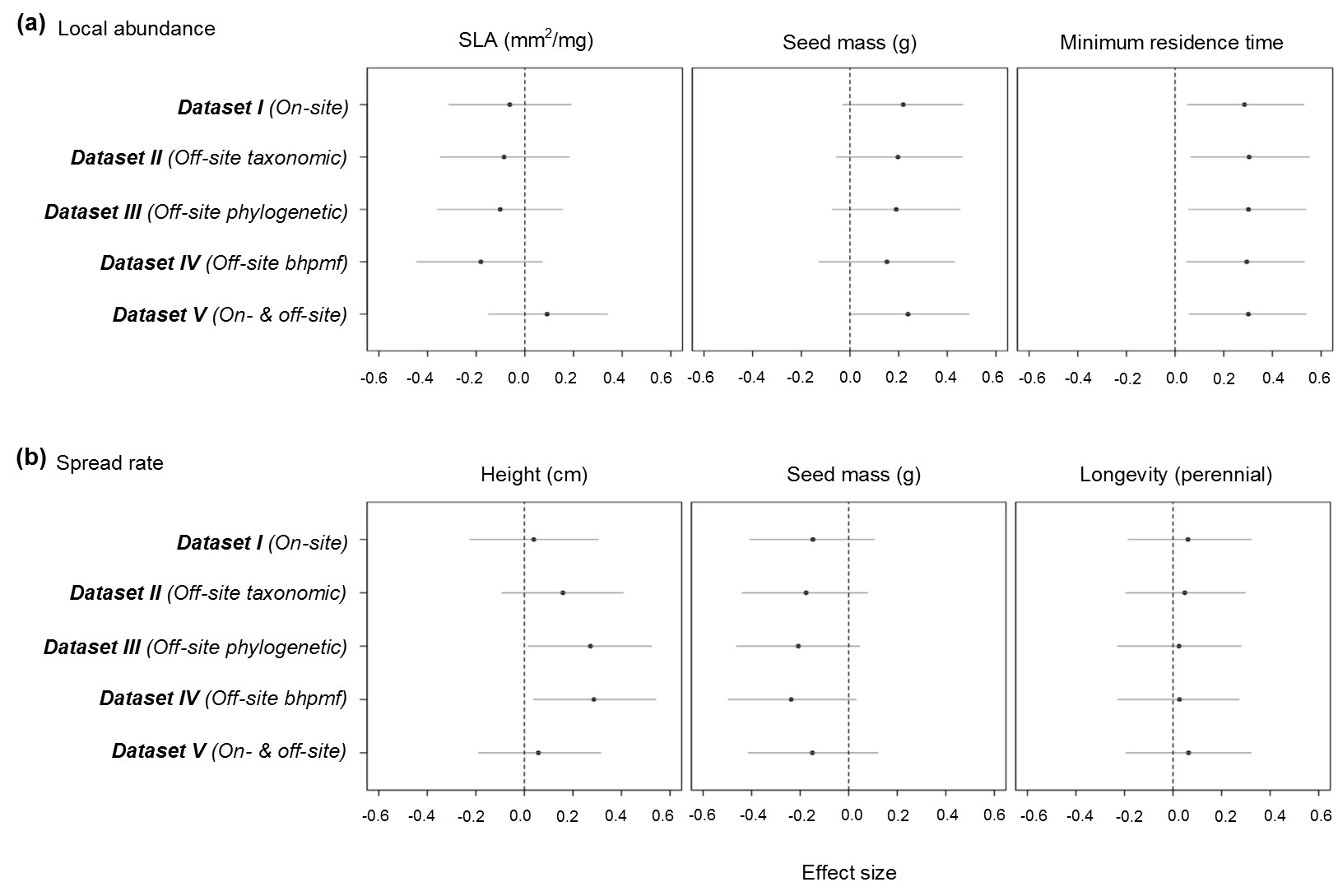


**Figure S9**. Effect of traits on exotic plants’ local abundance (a) and spread rate (b) when models are run using the same sample size (n=62 local abundance; n=67 spread rate). Dots and grey lines represent mean effects and 95% credible intervals, respectively. Unlike SLA and height, values of seed mass, minimum residence time and longevity did not change across datasets.

**
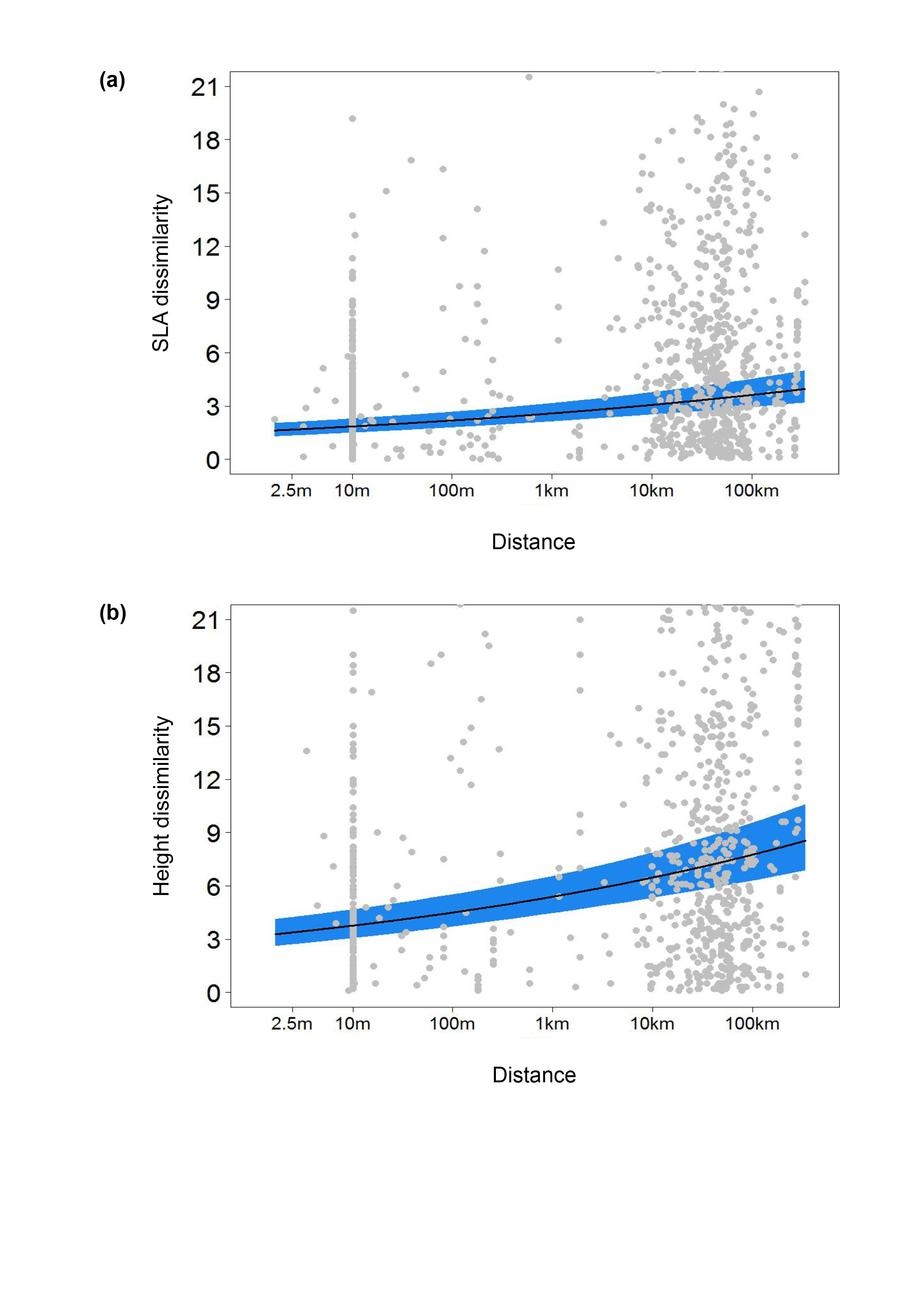
Figure S10.** Correlation between distance across pairs of samples of the same species and the (a) SLA or (b) height dissimilarity between the same pair of samples collected in Victoria, Australia. See Appendix S1 for more details.

**
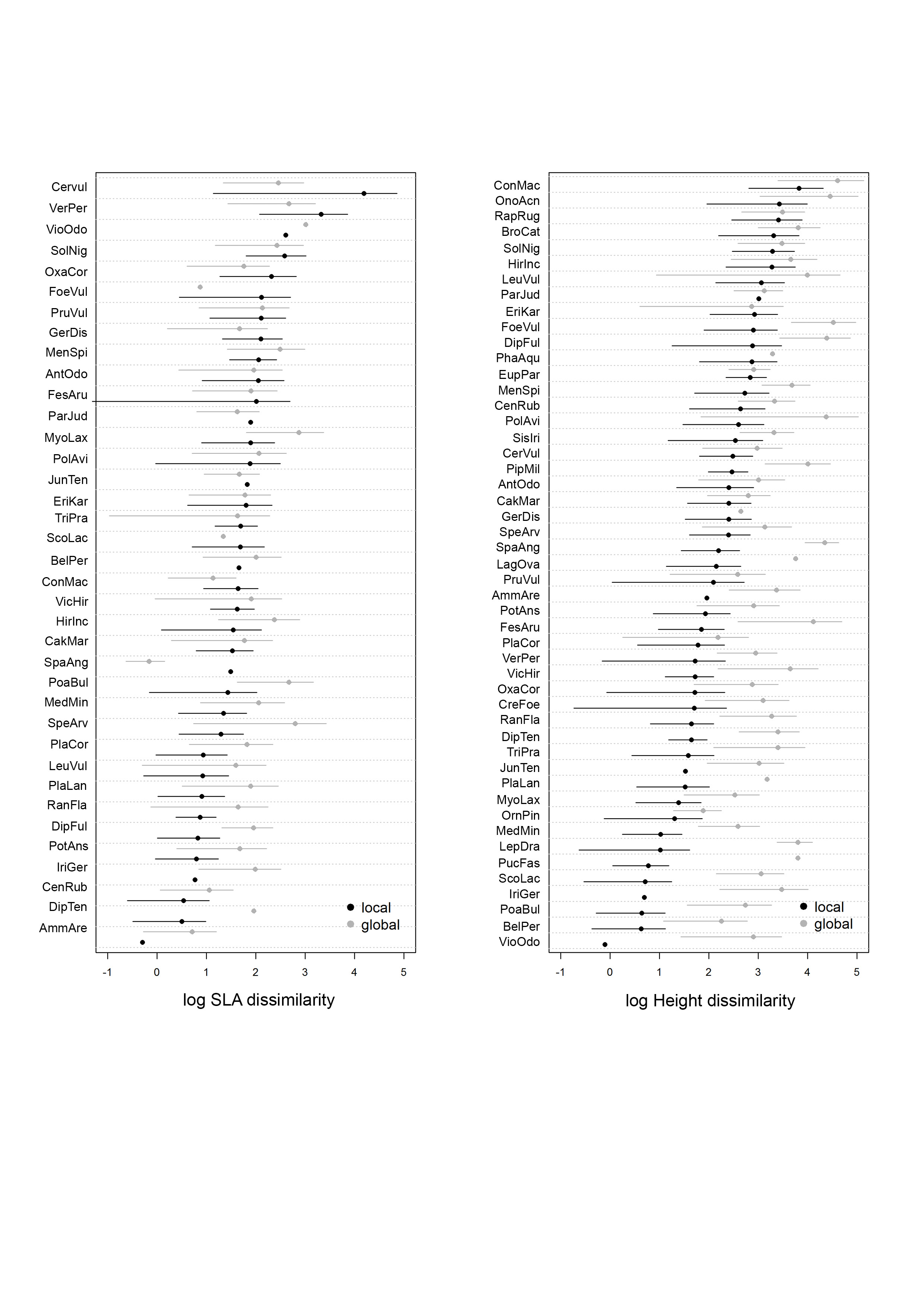
Figure S11.** Mean SLA (left) or height (right) dissimilarity across species’ records collected on-site (i.e. local; in black) or off-site (i.e. global; in grey). Dots and lines represent species’ means and standard deviations, respectively. Note x axis is log-transformed in both panels. Full species names provided in Table S2.


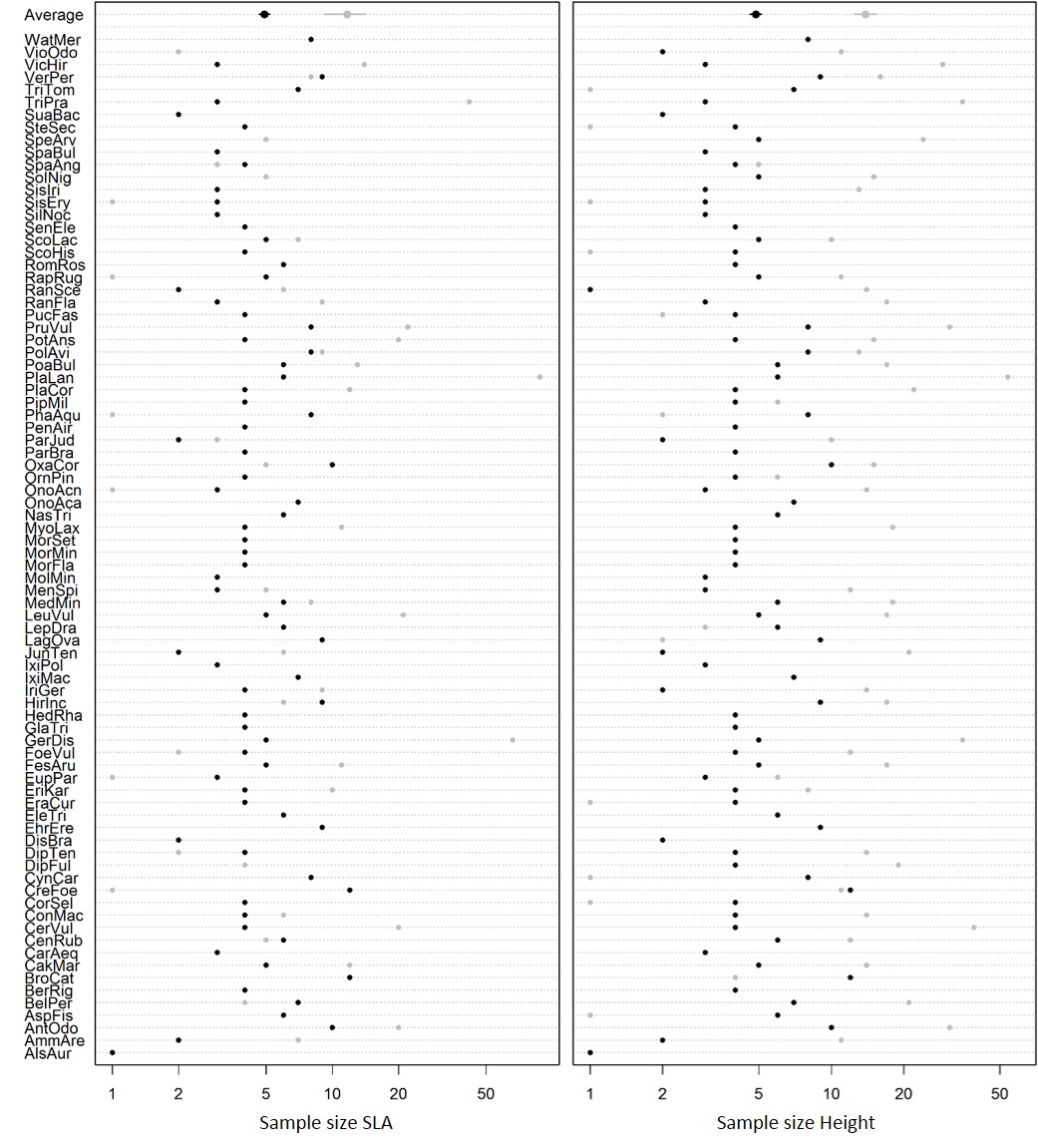
**Figure S12**. Number of samples collected on-site in Victoria (black dots) and off-site through the TRY database (grey dots) for 82 plant species in Victoria. Average and species-level sample size for SLA on the left panel and height on the right panel. Note the x-axis are presented in a logarithmic scale. Full species names provided in Table S2. Average number of SLA records (mean ± SE) on-site was 4.9 ± 0.3 and off-site was 11.7 ± 2.5; average number of height records (mean ± SE) on-site was 4.9 ± 0.3 and off-site was 13.9 ± 1.4.

**
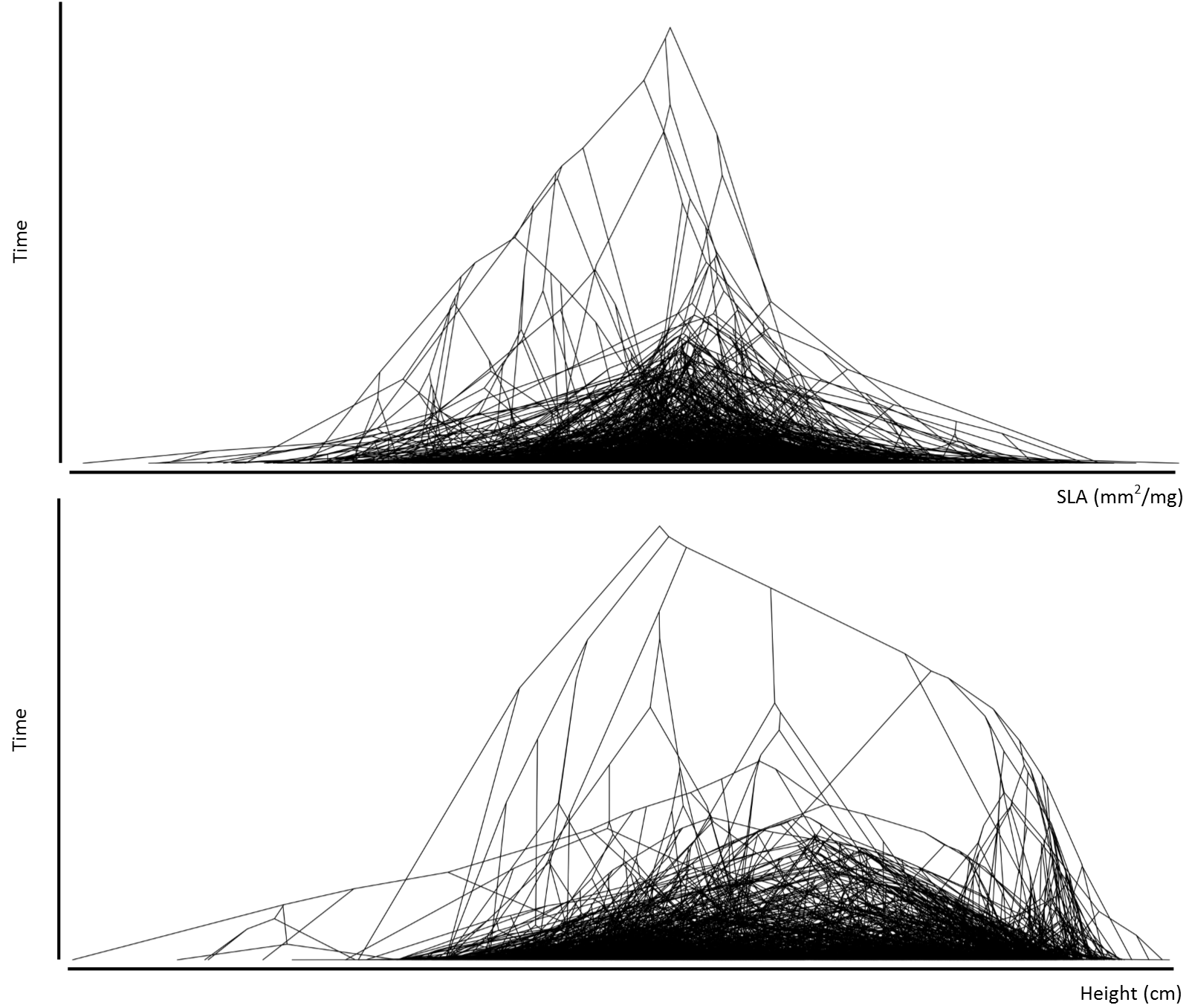
**

**Figure S13.** Traitgram of SLA and height showing the variability of trait values across clades, based on the public records from TRY used to run imputations for *Dataset II (Off-site data and taxonomic imputation)* and the phylogenetic tree published by Zanne et al. (2014).

**
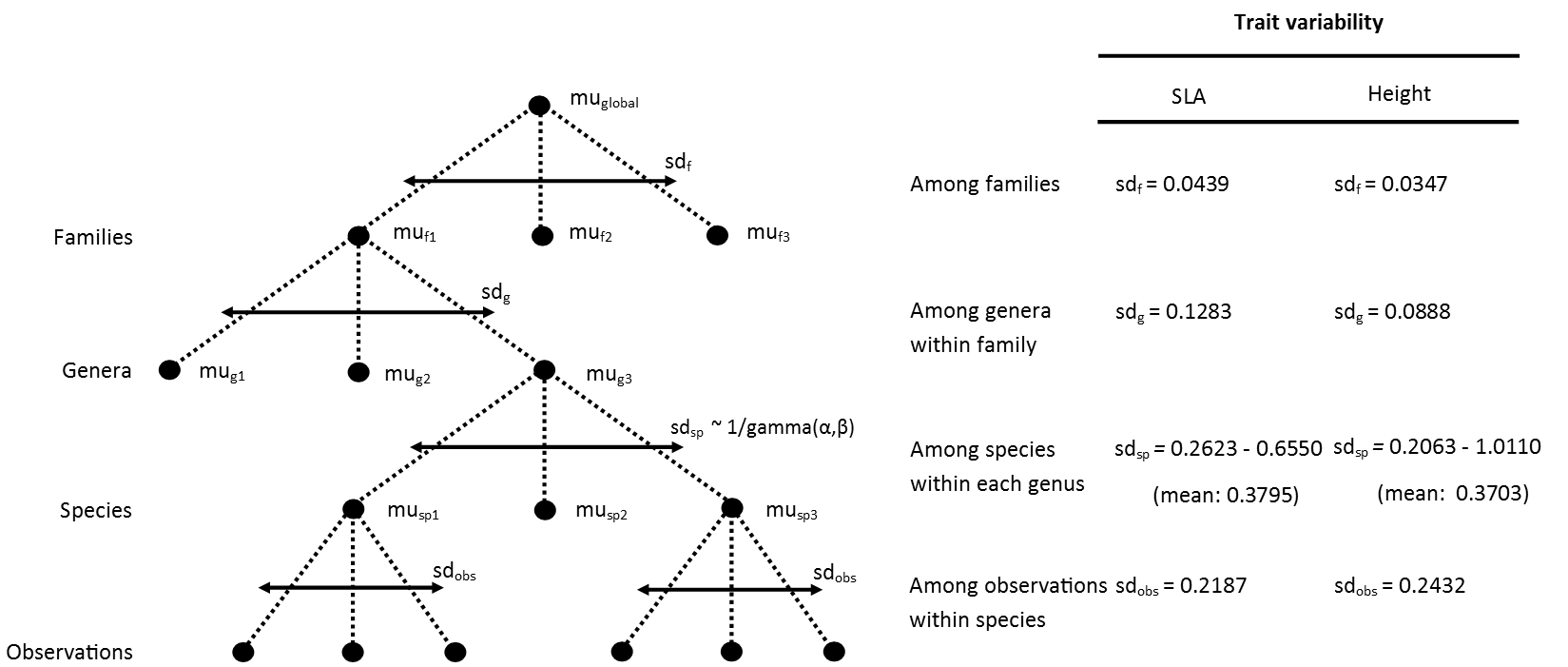
**

**Figure S14**. Representation of the structure of the taxonomy-nested hierarchical model used to run imputations for *Dataset II (Off-site data and taxonomic imputation)*. mu_sp_, mu_g_, mu_f_ and mu_global_ stand for the mean trait value of species s, genus g, family f and the whole dataset, respectively. sd_obs,_ sd_sp_, sd_g_ and sd_f_ stand for the variability across observations, species, genera and families, respectively. All the variability parameters were fixed, with the exception of sd_sp_ which was allowed to vary among genera following an inverse gamma distribution. Note that the highest trait variability was found among species within each genus (sd_sp_) for both SLA and height.

**REFERENCES**

Zanne A.E. et al. (2014) *Three keys to the radiation of angiosperms into freezing environments*. **Nature** 506: 89-92.
