# Supplementary material for: Building trait datasets: effect of methodological choice on a study of invasion
Source: Oecologia. 2022 Aug 17;199(4):919–35. doi: 10.1007/s00442-022-05230-8 (PMC9464113; doi:10.1007/s00442-022-05230-8)
Supplement: Supplementary file 2 — Supplementary file2 (DOCX 14002 kb) [file 442_2022_5230_MOESM2_ESM.docx]

**SUPPLEMENTARY MATERIAL – Tables S1 to S2**

**Palma et al. (2022) “Building trait datasets: effect of methodological choice on a study of invasion.” Oecologia**

**Table S1.** Sampling effort for trait collection in Victoria, Australia (*Dataset I – On-site data*), including total sample size per species, locations where they were found, number of samples collected from each sampling location, and minimum and maximum distance between samples. See Figure S1 for location of sampling sites in Victoria.

| **Plant species** | **N samples (total)** | **Sampling locations**  **(code and name)** | | **N samples**  **(per location)** | **Distance among samples (m, min)*** | **Distance among samples (m, max)** |
| --- | --- | --- | --- | --- | --- | --- |
| *Alstroemeria aurea* | 1 | **26** | Geelong North 2 | 1 | NA | NA |
| *Ammophila arenaria* | 2 | **17** | Frankston 1 | 2 | 6.4 | 6.4 |
| *Anthoxanthum odoratum* | 10 | **10** | Dandenongs 3 | 2 | 118.3 | 101,347.3 |
|  |  | **13** | Dandenongs 6 | 1 |  |  |
|  |  | **16** | Frankston 3 | 1 |  |  |
|  |  | **25** | Geelong North 3 | 1 |  |  |
|  |  | **28** | Frankston 7 | 1 |  |  |
|  |  | **46** | Yellingbo 1 | 1 |  |  |
|  |  | **48** | Yellingbo 3 | 1 |  |  |
|  |  | **58** | Long Forest 2 | 1 |  |  |
|  |  | **70** | Beaconsfield | 1 |  |  |
| *Asphodelus fistulosus* | 6 | **6** | Williamstown | 1 | 10.0 | 71,631.2 |
|  |  | **39** | Geelong South 3 | 2 |  |  |
|  |  | **41** | Altona 2 | 1 |  |  |
|  |  | **60** | Long Forest 4 | 1 |  |  |
|  |  | **67** | Mornington Penin 1 | 1 |  |  |
| *Bellis perennis* | 7 | **9** | Dandenongs 2 | 1 | 10.0 | 141,616.2 |
|  |  | **11** | Dandenongs 5 | 1 |  |  |
|  |  | **13** | Dandenongs 6 | 1 |  |  |
|  |  | **25** | Geelong North 3 | 1 |  |  |
|  |  | **71** | Warragul 2 | 3 |  |  |
| *Berkheya rigida* | 4 | **22** | Geelong North 5 | 1 | 191.4 | 55,210.3 |
|  |  | **37** | Geelong 1 | 1 |  |  |
|  |  | **45** | Canning Reserve | 2 |  |  |
| *Bromus catharticus* | 12 | **1** | Royal Park | 3 | 10.0 | 88,743.9 |
|  |  | **4** | Brimbank Park 1 | 1 |  |  |
|  |  | **6** | Williamstown | 1 |  |  |
|  |  | **7** | Altona 3 | 1 |  |  |
|  |  | **9** | Dandenongs 2 | 1 |  |  |
|  |  | **16** | Frankston 3 | 1 |  |  |
|  |  | **31** | Frankston 5 | 1 |  |  |
|  |  | **46** | Yellingbo 1 | 1 |  |  |
|  |  | **51** | Diamond Creek | 1 |  |  |
|  |  | **58** | Long Forest 2 | 1 |  |  |
|  |  |  |  |  |  |  |
| *Cakile maritima* | 5 | **90** | Soreham | 4 | 10.0 | 41,432.5 |
|  |  | **97** | Point Nepean | 1 |  |  |
| *Carpobrotus aequilaterus* | 3 | **92** | Lake Buloke | 3 | 10.0 | 10.0 |
| *Centranthus ruber* | 6 | **91** | Gippsland Lakes | 2 | 10.0 | 266,277.7 |
|  |  | **96** | Geelong 3 | 4 |  |  |
| *Cerastium vulgare* | 4 | **5** | Brimbank Park 2 | 1 | 10.0 | 53,224.7 |
|  |  | **54** | Kinglake 2 | 2 |  |  |
|  |  | **55** | Kinglake 1 | 1 |  |  |
| *Conium maculatum* | 4 | **4** | Brimbank Park 1 | 2 | 378.6 | 51,631.3 |
|  |  | **8** | Merry Creek | 1 |  |  |
|  |  | **24** | Geelong North 4 | 1 |  |  |
| *Cortaderia selloana* | 4 | **38** | Geelong South 2 | 2 | 27.8 | 58,264.0 |
|  |  | **41** | Altona 2 | 1 |  |  |
|  |  | **45** | Canning Reserve | 1 |  |  |
| *Crepis foetida* | 12 | **33** | Bendigo 3 | 2 | 16.0 | 128,257.9 |
|  |  | **36** | Geelong 4 | 1 |  |  |
|  |  | **40** | Geelong South 1 | 1 |  |  |
|  |  | **41** | Altona 2 | 1 |  |  |
|  |  | **43** | Craigieburn 3 | 1 |  |  |
|  |  | **46** | Yellingbo 1 | 1 |  |  |
|  |  | **48** | Yellingbo 3 | 1 |  |  |
|  |  | **51** | Diamond Creek | 1 |  |  |
|  |  | **54** | Kinglake 2 | 1 |  |  |
|  |  | **58** | Long Forest 2 | 1 |  |  |
|  |  | **61** | Andersons swamp 1 | 1 |  |  |
| *Cynara cardunculus* | 8 | **99** | Deer Park | 8 | 10.0 | 1,871.0 |
| *Dipsacus fullonum* | 4 | **79** | Ballarat 2 | 2 | 10.0 | 189,291.4 |
|  |  | **82** | Mount Eccles | 2 |  |  |
| *Diplotaxis tenuifolia* | 4 | **96** | Geelong 3 | 4 | 10.0 | 10.0 |
| *Disa bracteata* | 2 | **2** | Evans St | 2 | 43.4 | 43.4 |
| *Ehrharta erecta* | 9 | **1** | Royal Park | 2 | 10.0 | 95,785.9 |
|  |  | **4** | Brimbank Park 1 | 2 |  |  |
|  |  | **8** | Merry Creek | 1 |  |  |
|  |  | **25** | Geelong North 3 | 1 |  |  |
|  |  | **31** | Frankston 5 | 1 |  |  |
|  |  | **42** | Altona 1 | 1 |  |  |
|  |  | **49** | Blackburn | 1 |  |  |
| *Eleusine tristachya* | 6 | **77** | Wangaratta | 3 | 10.0 | 340,224.2 |
|  |  | **81** | Ballarat 1 | 2 |  |  |
|  |  | **83** | Warnambool | 1 |  |  |
|  |  |  |  |  |  |  |
| *Eragrostis curvula* | 4 | **6** | Williamstown | 1 | 291.4 | 80,079.4 |
|  |  | **65** | Yellow Gum | 2 |  |  |
|  |  | **66** | Mornington Penin 2 | 1 |  |  |
| *Erigeron karvinskianus* | 4 | **9** | Dandenongs 2 | 1 | 4565.8 | 87,681.0 |
|  |  | **12** | Dandenongs 4 | 1 |  |  |
|  |  | **13** | Dandenongs 6 | 1 |  |  |
|  |  | **27** | Geelong North 1 | 1 |  |  |
| *Euphorbia paralias* | 3 | **69** | Geelong South 4 | 3 | 94.7 | 213.5 |
| *Festuca arundinacea* | 5 | **20** | Point Wilson 2 | 1 | 6.7 | 96,712.5 |
|  |  | **32** | Bendigo 4 | 2 |  |  |
|  |  | **52** | Yarra Valley | 2 |  |  |
| *Foeniculum vulgare* | 4 | **37** | Geelong 1 | 1 | 22.0 | 74,537.0 |
|  |  | **43** | Craigieburn 3 | 2 |  |  |
|  |  | **59** | Long Forest 1 | 1 |  |  |
| *Geranium dissectum* | 5 | **6** | Williamstown | 1 | 135.3 | 54,755.0 |
|  |  | **8** | Merry Creek | 2 |  |  |
|  |  | **47** | Yellingbo 2 | 1 |  |  |
|  |  | **53** | Kinglake 3 | 1 |  |  |
| *Gladiolus tristis* | 4 | **95** | Paraparap | 4 | 10.0 | 10.0 |
| *Hedypnois rhagadioloides* | 4 | **61** | Andersons swamp 1 | 4 | 10.0 | 10.0 |
| *Hirschfeldia incana* | 9 | **43** | Craigieburn 3 | 3 | 1.7 | 189,892.2 |
|  |  | **45** | Canning Reserve | 1 |  |  |
|  |  | **47** | Yellingbo 2 | 1 |  |  |
|  |  | **52** | Yarra Valley | 1 |  |  |
|  |  | **57** | Long Forest 3 | 1 |  |  |
|  |  | **64** | Craigieburn 1 | 1 |  |  |
|  |  | **75** | Lake Glennmaggie | 1 |  |  |
| *Iris germanica* | 4 | **94** | Devil's kitchen | 4 | 10.0 | 10.0 |
| *Ixia maculata* | 7 | **85** | Jamieson | 4 | 10.0 | 283,261.1 |
|  |  | **87** | Ararat | 3 |  |  |
| *Ixia polystachya* | 3 | **49** | Blackburn | 3 | 10.0 | 10.0 |
| *Juncus tenuis* | 2 | **93** | Mortlake | 2 | 10.0 | 10.0 |
| *Lagurus ovatus* | 9 | **16** | Frankston 3 | 1 | 10.0 | 50,817.3 |
|  |  | **17** | Frankston 1 | 2 |  |  |
|  |  | **39** | Geelong South 3 | 2 |  |  |
|  |  | **41** | Altona 2 | 2 |  |  |
|  |  | **66** | Mornington Penin 2 | 2 |  |  |
| *Lepidium draba* | 6 | **21** | Point Wilson 1 | 2 | 10.0 | 81,663.6 |
|  |  | **36** | Geelong 4 | 2 |  |  |
|  |  | **42** | Altona 1 | 1 |  |  |
|  |  | **43** | Craigieburn 3 | 1 |  |  |
| *Leucanthemum vulgare* | 5 | **27** | Geelong North 1 | 1 | 12.2 | 145,811.7 |
|  |  | **46** | Yellingbo 1 | 2 |  |  |
|  |  | **72** | Warragul 1 | 2 |  |  |
| *Medicago minima* | 6 | **5** | Brimbank Park 2 | 1 | 6795.7 | 59,223.9 |
|  |  | **6** | Williamstown | 1 |  |  |
|  |  | **51** | Diamond Creek | 1 |  |  |
|  |  | **52** | Yarra Valley | 1 |  |  |
|  |  | **58** | Long Forest 2 | 1 |  |  |
|  |  | **63** | Keilor | 1 |  |  |
| *Mentha spicata* | 3 | **76** | Seimour | 3 | 10.0 | 208.6 |
| *Molineriella minuta* | 3 | **89** | Snape Reserve | 3 | 10.0 | 10.0 |
| *Moraea flaccida* | 4 | **87** | Ararat | 4 | 10.0 | 10.0 |
| *Moraea miniata* | 4 | **88** | Stewall | 4 | 10.0 | 10.0 |
| *Moraea setifolia* | 4 | **88** | Stewall | 4 | 10.0 | 10.0 |
| *Myosotis laxa* | 4 | **90** | Soreham | 4 | 10.0 | 10.0 |
| *Nassella trichotoma* | 6 | **3** | Brimbank | 1 | 3674.8 | 46,137.9 |
|  |  | **5** | Brimbank Park 2 | 1 |  |  |
|  |  | **20** | Point Wilson 2 | 1 |  |  |
|  |  | **41** | Altona 2 | 1 |  |  |
|  |  | **45** | Canning Reserve | 1 |  |  |
|  |  | **57** | Long Forest 3 | 1 |  |  |
| *Onopordum acaulon* | 7 | **86** | Mallee | 7 | 10.0 | 10.0 |
| *Onopordum acanthium* | 3 | **90** | Soreham | 3 | 10.0 | 10.0 |
| *Ornithopus pinnatus* | 4 | **19** | Frankston 4 | 4 | 10.0 | 1,159.5 |
| *Oxalis corniculata* | 10 | **4** | Brimbank Park 1 | 3 | 10.0 | 88,060.3 |
|  |  | **25** | Geelong North 3 | 1 |  |  |
|  |  | **41** | Altona 2 | 2 |  |  |
|  |  | **46** | Yellingbo 1 | 1 |  |  |
|  |  | **51** | Diamond Creek | 1 |  |  |
|  |  | **57** | Long Forest 3 | 1 |  |  |
|  |  | **62** | Andersons swamp 2 | 1 |  |  |
| *Paronychia brasiliana* | 4 | **74** | Lakes entrance 1 | 4 | 10.0 | 10.0 |
| *Parietaria judaica* | 2 | **8** | Merry Creek | 1 | 15524.7 | 15,524.7 |
|  |  | **49** | Blackburn | 1 |  |  |
| *Pentameris airoides* | 4 | **89** | Snape Reserve | 4 | 10.0 | 10.0 |
| *Phalaris aquatica* | 8 | **18** | Frankston 2 | 2 | 15.5 | 97,267.8 |
|  |  | **29** | Frankston 8 | 2 |  |  |
|  |  | **36** | Geelong 4 | 1 |  |  |
|  |  | **43** | Craigieburn 3 | 1 |  |  |
|  |  | **57** | Long Forest 3 | 1 |  |  |
|  |  | **64** | Craigieburn 1 | 1 |  |  |
| *Piptatherum miliaceum* | 4 | **100** | Darebin | 4 | 10.0 | 10.0 |
| *Plantago coronopus* | 4 | **6** | Williamstown | 1 | 1508.9 | 197,235.1 |
|  |  | **17** | Frankston 1 | 1 |  |  |
|  |  | **18** | Frankston 2 | 1 |  |  |
|  |  | **73** | Lakes entrance 2 | 1 |  |  |
| *Plantago lanceolata* | 6 | **4** | Brimbank Park 1 | 2 | 25.3 | 51,206.9 |
|  |  | **6** | Williamstown | 1 |  |  |
|  |  | **18** | Frankston 2 | 1 |  |  |
|  |  | **43** | Craigieburn 3 | 1 |  |  |
|  |  | **61** | Andersons swamp 1 | 1 |  |  |
| *Poa bulbosa* | 6 | **1** | Royal Park | 4 | 10.0 | 10.0 |
|  |  | **20** | Point Wilson 2 | 2 |  |  |
| *Polygonum aviculare* | 8 | **7** | Altona 3 | 1 | 10.0 | 268,012.7 |
|  |  | **21** | Point Wilson 1 | 1 |  |  |
|  |  | **36** | Geelong 4 | 2 |  |  |
|  |  | **44** | Craigieburn 2 | 1 |  |  |
|  |  | **63** | Keilor | 2 |  |  |
|  |  | **77** | Wangaratta | 1 |  |  |
| *Potentilla anserina* | 4 | **82** | Mount Eccles | 4 | 10.0 | 10.0 |
| *Prunella vulgaris* | 8 | **13** | Dandenongs 6 | 1 | 1687.0 | 189,350.1 |
|  |  | **14** | Dandenongs 7 | 1 |  |  |
|  |  | **26** | Geelong North 2 | 1 |  |  |
|  |  | **27** | Geelong North 1 | 1 |  |  |
|  |  | **46** | Yellingbo 1 | 1 |  |  |
|  |  | **48** | Yellingbo 3 | 1 |  |  |
|  |  | **56** | Coranderrk | 1 |  |  |
|  |  | **75** | Lake Glennmaggie | 1 |  |  |
| *Puccinellia fasciculata* | 5 | **21** | Point Wilson 1 | 5 | 10.0 | 10.0 |
| *Ranunculus flammula* | 3 | **48** | Yellingbo 3 | 3 | 10.0 | 300.5 |
| *Ranunculus sceleratus* | 2 | **84** | Granny's flat | 2 | 10.0 | 10.0 |
| *Rapistrum rugosum* | 5 | **6** | Williamstown | 2 | 625.1 | 82,272.9 |
|  |  | **21** | Point Wilson 1 | 1 |  |  |
|  |  | **36** | Geelong 4 | 1 |  |  |
|  |  | **44** | Craigieburn 2 | 1 |  |  |
| *Romulea rosea* | 6 | **84** | Granny's flat | 4 | 10.0 | 289,917.8 |
|  |  | **87** | Ararat | 2 |  |  |
| *Scolymus hispanicus* | 4 | **80** | Ballarat 3 | 4 | 30.6 | 151.5 |
| *Scorzonera laciniata* | 5 | **61** | Andersons swamp 1 | 4 | 10.0 | 14,566.3 |
|  |  | **63** | Keilor | 1 |  |  |
| *Senecio elegans* | 4 | **97** | Point Nepean | 4 | 10.0 | 10.0 |
| *Silene nocturna* | 3 | **74** | Lakes entrance 1 | 3 | 4.4 | 13.4 |
| *Sisymbrium erysimoides* | 3 | **35** | Bendigo 2 | 3 | 3.3 | 10.0 |
| *Sisymbrium irio* | 3 | **68** | Geelong 2 | 3 | 16.8 | 76.5 |
| *Solanum nigrum* | 5 | **8** | Merry Creek | 1 | 10.0 | 63,056.9 |
|  |  | **52** | Yarra Valley | 2 |  |  |
|  |  | **61** | Andersons swamp 1 | 1 |  |  |
|  |  | **70** | Beaconsfield | 1 |  |  |
| *Spartina anglica* | 4 | **98** | Dalmore | 4 | 10.0 | 10.0 |
| *Sparaxis bulbifera* | 3 | **85** | Jamieson | 3 | 10.0 | 10.0 |
| *Spergula arvensis* | 5 | **34** | Bendigo 1 | 3 | 10.0 | 280,934.1 |
|  |  | **73** | Lakes entrance 2 | 2 |  |  |
| *Stenotaphrum secundatum* | 4 | **67** | Mornington Penin 1 | 4 | 10.0 | 10.0 |
| *Suaeda baccifera* | 2 | **37** | Geelong 1 | 2 | 10.0 | 10.0 |
| *Trifolium pratense* | 3 | **50** | Wattle Park | 2 | 10.0 | 18,126.2 |
|  |  | **51** | Diamond Creek | 1 |  |  |
| *Trifolium tomentosum* | 7 | **78** | Cobram | 7 | 10.0 | 255.4 |
| *Veronica persica* | 9 | **4** | Brimbank Park 1 | 2 | 10.0 | 60,895.0 |
|  |  | **5** | Brimbank Park 2 | 1 |  |  |
|  |  | **13** | Dandenongs 6 | 2 |  |  |
|  |  | **47** | Yellingbo 2 | 2 |  |  |
|  |  | **51** | Diamond Creek | 2 |  |  |
| *Vicia hirsuta* | 3 | **23** | Gisborne | 1 | 52762.9 | 87,734.3 |
|  |  | **30** | Frankston 6 | 1 |  |  |
|  |  | **51** | Diamond Creek | 1 |  |  |
| *Viola odorata* | 2 | **8** | Merry Creek | 1 | 30089.6 | 30,089.6 |
|  |  | **10** | Dandenongs 3 | 1 |  |  |
| *Watsonia meriana* | 8 | **9** | Dandenongs 2 | 1 | 10.0 | 62,884.1 |
|  |  | **14** | Dandenongs 7 | 2 |  |  |
|  |  | **15** | Dandenongs 1 | 1 |  |  |
|  |  | **29** | Frankston 8 | 1 |  |  |
|  |  | **51** | Diamond Creek | 1 |  |  |
|  |  | **53** | Kinglake 3 | 2 |  |  |

* When multiple individuals in a sampling location were assigned the same geographic coordinates during fieldwork, we assumed they were 10m apart.

**Table S2.** Plant species included in *Datasets I* to *VI.* Available trait records are represented by *x*; imputed values are represented by *imp*.

|  | ***Dataset I*** *(On-site)* | ***Dataset II*** *(Off-site taxonomic)* | ***Dataset III*** *(Off-site phylogenetic)* | ***Dataset IV***  *(Off-site bhpmf)* | ***Dataset V*** *(On- & off-site)* | ***Dataset VI***  *(All off-site)* |
| --- | --- | --- | --- | --- | --- | --- |
| *Aira praecox* |  |  |  |  |  | x |
| *Allium vineale* |  |  |  |  |  | x |
| *Alstroemeria aurea* | x | imp^SLA,^ imp^HE^ | imp^SLA,^ imp^HE^ | imp^SLA,^ imp^HE^ | x |  |
| *Amaranthus deflexus* |  |  |  |  |  | x^HE^ |
| *Amaryllis belladonna* |  |  |  |  |  |  |
| *Ammophila arenaria* | x | x | x | x | x | x |
| *Anchusa arvensis* |  |  |  |  |  | x |
| *Anthoxanthum odoratum* | x | x | x | x | x | x |
| *Aponogeton distachyos* |  |  |  |  |  |  |
| *Arrhenatherum elatius* |  |  |  |  |  | x |
| *Artemisia verlotiorum* |  |  |  |  |  | x^HE^ |
| *Asphodelus fistulosus* | x | x^HE^, imp^SLA^ | x^HE^ | x^HE^, imp^SLA^ | x | x^HE^ |
| *Bellis perennis* | x | x | x | x | x | x |
| *Berkheya rigida* | x | imp^SLA^, imp^HE^ | imp^SLA^, imp^HE^ | imp^SLA^, imp^HE^ | x |  |
| *Beta vulgaris* |  |  |  |  |  | x |
| *Bromus catharticus* | x | x^HE^, imp^SLA^ | x^HE^, imp^SLA^ | x^HE^, imp^SLA^ | x | x^HE^ |
| *Buglossoides arvensis* |  |  |  |  |  | x^HE^ |
| *Cakile maritima* | x | x | x | x | x | x |
| *Cardamine hirsuta* |  |  |  |  |  | x |
| *Carpobrotus aequilaterus* | x | imp^SLA^, imp^HE^ |  | imp^SLA^, imp^HE^ | x |  |
| *Cenchrus clandestinus* |  |  |  |  |  |  |
| *Cenchrus longisetus* |  |  |  |  |  |  |
| *Cenchrus longispinus* |  |  |  |  |  |  |
| *Cenchrus macrourus* |  |  |  |  |  |  |
| *Centaurea nigra* |  |  |  |  |  | x |
| *Centaurea solstitialis* |  |  |  |  |  | x |
| *Centranthus ruber* subsp*. ruber* | x | x | x | x | x | x |
| *Cerastium comatum* |  |  |  |  |  |  |
| *Cerastium vulgare* | x | x | x | x | x | x |
| *Chamaemelum nobile* |  |  |  |  |  | x^HE^ |
| *Citrullus lanatus* |  |  |  |  |  | x^HE^ |
| *Conium maculatum* | x | x | x | x | x | x |
| *Cortaderia selloana* | x | x^HE^, imp^SLA^ | x^HE^ | x^HE^, imp^SLA^ | x | x^HE^ |
| *Crepis foetida* subsp*. foetida* | x | x | x | x | x | x |
| *Crepis vesicaria* |  |  |  |  |  | x |
| *Crocosmia* x *crocosmiiflora* |  |  |  |  |  |  |
| *Cynara cardunculus* | x | x^HE^, imp^SLA^ | x^HE^, imp^SLA^ | x^HE^, imp^SLA^ | x | x^HE^ |
| *Cyperus congestus* |  |  |  |  |  |  |
| *Danthonia decumbens* |  |  |  |  |  | x |
| *Dianthus armeria* |  |  |  |  |  | x |
| *Digitalis purpurea* |  |  |  |  |  | x |
| *Diplotaxis tenuifolia* | x | x | x | x | x | x |
| *Dipsacus fullonum* | x | x | x | x | x | x |
|  | ***Dataset I*** *(On-site)* | ***Dataset II*** *(Off-site taxonomic)* | ***Dataset III*** *(Off-site phylogenetic)* | ***Dataset IV***  *(Off-site bhpmf)* | ***Dataset V*** *(On- & off-site)* | ***Dataset VI***  *(All off-site)* |
| *Disa bracteata* | x | imp^SLA^, imp^HE^ | imp^SLA^, imp^HE^ | imp^SLA^, imp^HE^ | x |  |
| *Dysphania multifida* |  |  |  |  |  |  |
| *Ehrharta erecta* var*. erecta* | x | imp^SLA^, imp^HE^ | imp^SLA^, imp^HE^ | imp^SLA^, imp^HE^ | x |  |
| *Eleusine tristachya* | x | imp^SLA^, imp^HE^ | imp^SLA^, imp^HE^ | imp^SLA^, imp^HE^ | x |  |
| *Elymus farctus* |  |  |  |  |  | x |
| *Epilobium ciliatum* |  |  |  |  |  | x |
| *Epilobium hirsutum* |  |  |  |  |  | x |
| *Eragrostis curvula* | x | x^HE^, imp^SLA^ | x^HE^, imp^SLA^ | x^HE^, imp^SLA^ | x | x^HE^ |
| *Eragrostis mexicana* |  |  |  |  |  |  |
| *Erigeron karvinskianus* | x | x | x | x | x | x |
| *Erodium brachycarpum* |  |  |  |  |  |  |
| *Euphorbia lathyris* |  |  |  |  |  | x |
| *Euphorbia paralias* | x | x | x | x | x | x |
| *Festuca arundinacea* | x | x | x | x | x | x |
| *Foeniculum vulgare* | x | x | x | x | x | x |
| *Freesia alba* x *leichtlinii* |  |  |  |  |  |  |
| *Gastridium phleoides* |  |  |  |  |  |  |
| *Gaudinia fragilis* |  |  |  |  |  | x^HE^ |
| *Geranium dissectum* | x | x | x | x | x | x |
| *Gladiolus tristis* | x | imp^SLA^, imp^HE^ | imp^HE^ | imp^SLA^, imp^HE^ | x |  |
| *Glaucium flavum* |  |  |  |  |  | x |
| *Glyceria declinata* |  |  |  |  |  | x |
| *Hainardia cylindrica* |  |  |  |  |  |  |
| *Hedypnois rhagadioloides* | x | imp^SLA^, imp^HE^ | imp^SLA^, imp^HE^ | imp^SLA^, imp^HE^ | x |  |
| *Heliotropium supinum* |  |  |  |  |  |  |
| *Hirschfeldia incana* | x | x | x | x | x | x |
| *Holcus annuus* |  |  |  |  |  |  |
| *Hypericum tetrapterum* |  |  |  |  |  | x |
| *Iris germanica* | x | x | x | x | x | x |
| *Isolepis hystrix* |  |  |  |  |  |  |
| *Ixia maculata* | x | imp^SLA^, imp^HE^ |  | imp^SLA^, imp^HE^ | x |  |
| *Ixia polystachya* | x | imp^SLA^, imp^HE^ |  | imp^SLA^, imp^HE^ | x |  |
| *Juncus tenuis* | x | x | x | x | x | x |
| *Kickxia elatine* |  |  |  |  |  | x |
| *Lagurus ovatus* | x | x^HE^, imp^SLA^ | x^HE^, imp^SLA^ | x^HE^, imp^SLA^ | x | x^HE^ |
| *Lamarckia aurea* |  |  |  |  |  | x^HE^ |
| *Lamium amplexicaule* |  |  |  |  |  | x |
| *Lapsana communis* |  |  |  |  |  |  |
| *Lepidium draba* | x | x^HE^, imp^SLA^ | x^HE^, imp^SLA^ | x^HE^, imp^SLA^ | x | x^HE^ |
| *Leucanthemum vulgare* | x | x | x | x | x | x |
| *Lilaea scilloides* |  |  |  |  |  | x^HE^ |
| *Ludwigia palustris* |  |  |  |  |  | x |
| *Malva arborea* |  |  |  |  |  | x^HE^ |
| *Medicago minima* | x | x | x | x | x | x |
| *Melissa officinalis* |  |  |  |  |  | x^HE^ |
| *Mentha spicata* | x | x | x | x | x | x |
| *Mimulus moschatus* |  |  |  |  |  | x |
|  | ***Dataset I*** *(On-site)* | ***Dataset II*** *(Off-site taxonomic)* | ***Dataset III*** *(Off-site phylogenetic)* | ***Dataset IV***  *(Off-site bhpmf)* | ***Dataset V*** *(On- & off-site)* | ***Dataset VI***  *(All off-site)* |
| *Molineriella minuta* | x | imp^SLA^, imp^HE^ |  |  | x |  |
| *Moraea flaccida* | x | imp^SLA^, imp^HE^ |  | imp^SLA^, imp^HE^ | x |  |
| *Moraea miniata* | x | imp^SLA^, imp^HE^ |  | imp^SLA^, imp^HE^ | x |  |
| *Moraea setifolia* | x | imp^SLA^, imp^HE^ |  | imp^SLA^, imp^HE^ | x |  |
| *Myosotis laxa* subsp*. caespitosa* | x | x | x | x | x | x |
| *Myriophyllum aquaticum* |  |  |  |  |  | x^HE^ |
| *Nassella trichotoma* | x | imp^SLA^, imp^HE^ | imp^HE^ | imp^SLA^, imp^HE^ | x |  |
| *Onopordum acanthium* subsp*. acanthium* | x | x | x | x | x | x |
| *Onopordum acaulon* | x | imp^SLA^, imp^HE^ | x | imp^SLA^, imp^HE^ | x |  |
| *Onopordum illyricum* |  |  |  |  |  |  |
| *Ornithopus pinnatus* | x | x^HE^, imp^SLA^ | x^HE^, imp^SLA^ | x^HE^, imp^SLA^ | x | x^HE^ |
| *Oxalis corniculata* s.s. | x | x | x | x | x | x |
| *Panicum capillare* |  |  |  |  |  | x |
| *Panicum coloratum* |  |  |  |  |  | x^HE^ |
| *Papaver hybridum* |  |  |  |  |  | x^HE^ |
| *Parietaria judaica* | x | x | x | x | x | x |
| *Paronychia brasiliana* | x | imp^SLA^, imp^HE^ |  | imp^SLA^, imp^HE^ | x |  |
| *Pentameris airoides* | x | imp^SLA^, imp^HE^ | imp^SLA^ | imp^SLA^, imp^HE^ | x |  |
| *Phalaris aquatica* | x | x | x | x | x | x |
| *Piptatherum miliaceum* | x | x^HE^, imp^SLA^ | x^HE^ | x^HE^, imp^SLA^ | x | x^HE^ |
| *Plantago bellardii* |  |  |  |  |  | x^HE^ |
| *Plantago coronopus* | x | x | x | x | x | x |
| *Plantago lanceolata* | x | x | x | x | x | x |
| *Poa bulbosa* | x | x | x | x | x | x |
| *Polygonum aviculare* s.s. | x | x | x | x | x | x |
| *Potentilla anserina* | x | x | x | x | x | x |
| *Potentilla indica* |  |  |  |  |  |  |
| *Prunella vulgaris* | x | x | x | x | x | x |
| *Psilurus incurvus* |  |  |  |  |  | x^HE^ |
| *Puccinellia fasciculata* | x | x^HE^, imp^SLA^ | x^HE^, imp^SLA^ | x^HE^, imp^SLA^ | x | x^HE^ |
| *Ranunculus flammula* subsp*. flammula* | x | x | x | x | x | x |
| *Ranunculus ophioglossifolius* |  |  |  |  |  | x^HE^ |
| *Ranunculus sceleratus* | x | x | x | x | x | x |
| *Rapistrum rugosum* | x | x | x | x | x | x |
| *Romulea rosea* | x | imp^SLA^, imp^HE^ |  | imp^SLA^, imp^HE^ | x |  |
| *Rostraria pumila* |  |  |  |  |  |  |
| *Sagina maritima* |  |  |  |  |  | x |
| *Salpichroa origanifolia* |  |  |  |  |  | x^HE^ |
| *Saponaria officinalis* |  |  |  |  |  | x |
| *Scolymus hispanicus* | x | x^HE^, imp^SLA^ | x^HE^, imp^SLA^ | x^HE^, imp^SLA^ | x | x^HE^ |
| *Scorzonera laciniata* | x | x | x | x | x | x |
| *Sedum praealtum* |  |  |  |  |  |  |
| *Senecio elegans* | x | imp^SLA^, imp^HE^ | imp^SLA^, imp^HE^ | imp^SLA^, imp^HE^ | x |  |
| *Senecio vulgaris* |  |  |  |  |  | x |
|  | ***Dataset I*** *(On-site)* | ***Dataset II*** *(Off-site taxonomic)* | ***Dataset III*** *(Off-site phylogenetic)* | ***Dataset IV***  *(Off-site bhpmf)* | ***Dataset V*** *(On- & off-site)* | ***Dataset VI***  *(All off-site)* |
| *Silene nocturna* | x | imp^SLA^, imp^HE^ |  | imp^SLA^, imp^HE^ | x |  |
| *Sisymbrium erysimoides* | x | x | x | x | x | x |
| *Sisymbrium irio* | x | x^HE^, imp^SLA^ | x^HE^ | x^HE^, imp^SLA^ | x | x^HE^ |
| *Solanum chenopodioides* |  |  |  |  |  | x^HE^ |
| *Solanum nigrum* s.s. | x | x | x | x | x | x |
| *Sparaxis bulbifera* | x | imp^SLA^, imp^HE^ |  | imp^SLA^, imp^HE^ | x |  |
| *Spartina anglica* | x | x | x | x | x | x |
| *Spergula arvensis* | x | x | x | x | x | x |
| *Stenotaphrum secundatum* | x | x^HE^, imp^SLA^ | x^HE^, imp^SLA^ | x^HE^, imp^SLA^ | x | x^HE^ |
| *Suaeda baccifera* | x | imp^SLA^, imp^HE^ |  |  | x |  |
| *Tribolium acutiflorum* |  |  |  |  |  |  |
| *Tribolium obliterum* |  |  |  |  |  |  |
| *Trifolium pratense* | x | x | x | x | x | x |
| *Trifolium tomentosum* var*. tomentosum* | x | x^HE^, imp^SLA^ | x^HE^, imp^SLA^ | x^HE^, imp^SLA^ | x | x^HE^ |
| *Typha latifolia* |  |  |  |  |  | x |
| *Verbena supina* |  |  |  |  |  |  |
| *Veronica persica* | x | x | x | x | x | x |
| *Vicia hirsuta* | x | x | x | x | x | x |
| *Viola arvensis* |  |  |  |  |  | x |
| *Viola odorata* | x | x | x | x | x | x |
| *Watsonia meriana* var*. bulbillifera* | x | imp^SLA^, imp^HE^ |  | imp^SLA^, imp^HE^ | x |  |
| *Watsonia versfeldii* |  |  |  |  |  |  |
| *Zaluzianskya divaricata* |  |  |  |  |  |  |
