# Supplementary material for: Building trait datasets: effect of methodological choice on a study of invasion
Source: Oecologia. 2022 Aug 17;199(4):919–35. doi: 10.1007/s00442-022-05230-8 (PMC9464113; doi:10.1007/s00442-022-05230-8)
Supplement: Supplementary file 3 — Supplementary file3 (DOCX 51 kb) [file 442_2022_5230_MOESM3_ESM.docx]

**ELECTRONIC SUPPLEMENTARY MATERIAL – Appendix S1 to S4**

**Palma et al. (2022) “Building trait datasets: effect of methodological choice on a study of invasion.” Oecologia**

**Appendix S1. Extended information for on-site collection in Victoria, Australia**

Traits of 82 plant species were measured in Victoria. Vegetative height was measured for an average of five individuals per species and leaf samples for the same individuals were collected. Once collected, these samples were taken to the lab to estimate their specific leaf area. All the individuals collected in Victoria were healthy, mature plants growing in, to a greater or lesser degree, disturbed environments, from road verges to walking trails, and as far as possible only individuals growing under full sun exposure were measured (Perez-Harguindeguy *et al.* 2013). As far as possible for each species, individuals coming from different populations were collected. Attempts were made to collect records from a range of locations and habitats as wide as possible for all the species (Table S1). However, this was not always feasible and for some species we could only collect records from single populations. Sampling locations in Victoria are shown in Figure S1. None of these locations contained all the 82 plant species.

Vegetative height of whole individuals was measured in the field (before samples for SLA were collected) as the shortest distance between the upper boundary of the plant’s main photosynthetic tissues (i.e. the foliage excluding inflorescences) and the ground level. Specific leaf area was estimated as the one-sided area of fresh leaves divided by the oven-dried weight of those leaves. For each individual, the one-sided area of an average of 3 fresh leaves was measured using ImageJ software. The youngest fully expanded leaves were selected and their petiole and bracts (blade only for grasses) were included in the measurement. These samples were then transferred to an oven, dried for 72 h at 70 ˚C, and weighted.

We explored the correlation between trait intraspecific variability and geographic scale for the 82 plant species found in Victoria. First, we calculated the geographic distance between pairs of samples of each species in QGIS v.3.16.3. Second, we calculated the absolute difference in both SLA and height values between the same pairs of samples using function *dist()* (method=”euclidean”) of R package *stats*. Then, we run a linear model to estimate the correlation between geographic distance, *Geographic_distance*, and trait dissimilarity for each trait, *Trait_dissimilarity*, using species as random effect:

Trait_dissimilarity ~ dnorm(µ_i_, σ^-2^)

mu_i_ <- α_i_ + β * Geographic_distance

α_i_ ~ dnorm(µ_α_, σ_α_^-2^)

where *i* represents each plant species, and both the dissimilarity in the trait values and the distance between samples are log-transformed and standardised. µ_α_ and β were assigned a normal prior with mean=0 and sd=0.0001; σ_α_ and σ were assigned uniform priors with mean=0, and sd=100 and sd=5, respectively. The models were built in R (R Core Team, 2020) using a Bayesian inference framework through *R2jags* package (Yu-Sung & Masanao, 2020). For each model, we run 3 chains, and retained 10,000 iterations after 2,000 burn-in iterations.

|  | SLA | | Height | |
| --- | --- | --- | --- | --- |
|  | mean | sd | mean | sd |
| β | 0.204 | 0.027 | 0.213 | 0.026 |
| µ_α_ | -0.045 | 0.038 | 0.005 | 0.037 |
| σ_α_ | 0.307 | 0.029 | 0.309 | 0.029 |
| σ | 0.400 | 0.007 | 0.387 | 0.006 |

We found that the dissimilarity of traits between two samples of the same species increased with the distance between the samples (Fig. S10). Overall, individuals that were collected 10 metres, 1 kilometre and 10 kilometres apart showed differences of 1.8, 2.6 and 3.1 mm2/mg in their SLA values, respectively. Overall, individuals that were collected 10 metres, 1 kilometre and 10 kilometres apart showed differences of 3.8, 5.4 and 6.5 cm in their height values, respectively.

**Appendix S2. Code for statistical analyses**

**Imputation of missing species-level mean trait values for *Dataset II – Off-site data and taxonomic imputation*.**

global.records: data frame with all available records of trait t for non-woody plant species within the acceptable environmental criteria (Appendix S3). It includes columns for: Accepted_Species_Name (as a factor), Genus (as a factor), Family (as a factor) and Trait_value (as numeric, log-transformed and standardized). Species, genera and families with a single record are removed to improve model convergence. Those species (from the original 82 evaluated) with no species-level records in TRY show a missing value (i.e. NA) for the Trait_value column.

### JAGS CODE

cat(' model{

for (i in 1:nobs){ # Observations

stdz.log.trait[i] ~ dnorm (MUS[s[i]], pow(SIGMA,-2))

}

for (i in 1:nsp){ # Species level

MUS[i] ~ dnorm (MUG[g[i]], TAUS[g[i]])

}

for (i in 1:ngen){ # Genus level

MUG[i] ~ dnorm (MUF[f[i]], TAUG)

TAUS[i] ~ dgamma (alphaG, betaG)

}

for (i in 1:nfam){ # Family level

MUF[i] ~ dnorm (MUglobal, pow(SIGMAF,-2))

}

# Priors

MUglobal ~ dnorm (0, 0.001)

SIGMA ~ dunif (0,5)

SIGMAF ~ dunif (0,5)

TAUG ~ dgamma (0.01,0.01)

alphaG ~ dexp (1)

betaG ~ dgamma (0.001,0.001)

# Derived quantities

SIGMAS <- sqrt (1/TAUS)

SIGMAG <- sqrt (1/TAUG)

} '

, file = (modelfile <- tempfile( )))

jags.data <- list (stdz.log.trait=as.numeric(global.records$Trait_value),

nobs=as.numeric(dim(global.records)[1]), nsp=as.numeric(length(levels(as.factor(global.records$Accepted_Species_Name)))),

ngen=as.numeric(length(levels(global.records$Genus))),

nfam=as.numeric(length(levels(global.records$Family))),

s=as.numeric(global.records$Accepted_Species_Name),

g=as.numeric(global.records$Genus),

f=as.numeric(global.records$Family))

inits <- function () list(MUglobal=rnorm(1), SIGMA=runif(1), SIGMAF=runif(1), TAUG=1, alphaG=1, betaG=1)

parameters <- c ("SIGMA", "MUS", "SIGMAS", "MUG", "SIGMAG", "MUF", "MUglobal", "SIGMAF", "alphaG", "betaG")

n.chains=3

n.iter=50000

n.burnin=10000

out <- jags (data = jags.data, inits = inits, parameters.to.save = parameters, model.file = modelfile, n.chains=n.chains, n.iter=n.iter, n.burnin=n.burnin)

# Parameter MUS is the estimated mean trait for each species

**Imputation of missing species-level mean trait values for *Dataset III – Off-site data with phylogenetic imputation*.**

Zanne.tree <- read.tree (file="PhylogeneticResourcesVascular_Plants_rooted_dated.tre") # from Zanne et al 2014

my.tree <- read.newick(file="tree_82sp") # Tree with my species

chronogram.my.tree <- chronos(my.tree) # Transform into ultrametric tree (distance from root to every tip to be the same in millions of years)

# Check that species names in chronogram.my.tree are the same as in Zanne.tree

mean.global.records: data frame with species-level mean trait values for all the species in global.records. In its original units (i.e. not transformed or standardised). Row names are set to match species names. Build one data frame for each trait.

# Check that species names in mean.global.records are the same as in the previous trees

library("phyndr")

phyndr.trait <- phyndr_topology(chronogram.my.tree,

rownames(mean.global.records), Zanne.tree)

# Because multiple possible swaps are given by the previous function, we estimate the posterior mean and sd of the trait value after 1000 iterations of randomly picking one species out of all the possible swaps

phyndr.trait.post <- data.frame (Accepted_Species_Name=NA, mean.trait=NA,

sd.trait = NA)

for (i in 1: length (phyndr.trait$clades)) {

swaps <- mean.global.records [mean.global.records$Accepted_Species_Name

%in% phyndr.trait$clades[[i]],]

swaps1000 <- sample (swaps$mean.trait, 1000, replace=TRUE)

swaps1000.post <- data.frame (Accepted_Species_Name=

names(phyndr.trait$clades[i]),

mean.SLA = mean(swaps1000),

sd.SLA = sd(swaps1000))

phyndr.trait.post <- rbind (phyndr.trait.post, swaps1000.post)

rm (swaps, swaps1000, swaps1000.post)

}

phyndr.trait.post <- phyndr.trait.post[-1,]

**Imputation of missing species-level mean trait values for *Dataset IV – Off-site data with bhpmf imputation*.**

trait.records: data matrix with individual-level trait records for all the species in global.records. Traits as columns, with their values log-transformed and standardised. Individuals must have records for at least one trait, and NAs for the other columns. Species with missing values for trait t will show as NAs in the data matrix, which will then be imputed. Row names are set to match species names.

hierarchy.records: data frame with columns Accepted_Species_Name, Genus, Family, Order and Group for the same rows (individuals) as previous object trait.records.

library(devtools)

install_github("fisw10/BHPMF")

library(BHPMF)

GapFilling (trait.records, hierarchy.records,

prediction.level = 5,

used.num.hierarchy.levels = 4,

rmse.plot.test.data = TRUE,

mean.gap.filled.output.path = paste0(tmp.dir,"/mean_gap_filled.txt"),

std.gap.filled.output.path = paste0(tmp.dir,"/std_gap_filled.txt"),

tmp.dir=tmp.dir)

**Bayesian update to estimate *Dataset V – On- & off-site data***

my.priors: data frame containing the posterior mean and sd of parameter MUS as estimated for *Dataset II – Off-site data and taxonomic imputation* (see previous section ‘Imputation of missing species-level mean trait values for *Dataset II – Off-site data and taxonomic imputation’*) for the original 82 species evaluated.

my.records: data frame with the records of trait t collected in Victoria, for the original 82 species evaluated. It includes columns for: Accepted_Species_Name (as a factor) and Trait_value (as numeric, log-transformed and standardized).

### JAGS CODE

cat(' model{

for (i in 1:nobsVIC){

local.trait[i] ~ dnorm (mu[s[i]], pow(sigma,-2))

}

# Priors

for (i in 1:nspVIC){

mu[i] ~ dnorm (mus[i], pow(sigmas[i],-2))

}

sigma ~ dunif(0,100)

} '

, file = (modelfile <- tempfile()))

jags.data <- list(local.trait=as.numeric(my.records$Trait_value),

nobsVIC=as.numeric(dim(my.records)[1]), nspVIC=as.numeric(length(levels(my.records$Accepted_Species_Name))),

s=as.numeric(my.records$Accepted_Species_Name),

mus=my.priors$mean,

sigmas=my.priors$sd)

inits <- function () list(sigma=runif(1))

parameters <- c ("mu","sigma")

n.chains=3

n.iter=10000

n.burnin=2000

out <- jags (data = jags.data, inits = inits, parameters.to.save = parameters, model.file = modelfile, n.chains=n.chains, n.iter=n.iter, n.burnin=n.burnin)

**Invasiveness models**

stdz.log.inv.metric: vector of log-transformed (for spread rate)/logit-transformed (for local abundance) and standardized values of the invasiveness metric

stdz.log.trait: vector of log-transformed and standardized values of trait t

### JAGS CODE

cat(' model{

for (i in 1:Ntaxa){

stdz.log.inv.metric [i] ~ dnorm (mu[i], pow(sd,-2))

mu[i] <- alpha + beta1 * stdz.log.trait1[i]

+ beta2 * stdz.log.trait2[i]

+ beta3 * stdz.log.trait3[i]

}

# Predicted & Residuals

for (i in 1:Ntaxa){

residual[i] <- stdz.log.inv.metric[i] - mu[i] # Residuals for observed data

predicted[i] <- mu[i] # Predicted values

}

# Priors

alpha ~ dnorm (0, 0.001)

beta1 ~ dnorm (0, 0.001)

beta2 ~ dnorm (0, 0.001)

beta3 ~ dnorm (0, 0.001)

sd ~ dunif (0, 5)

} '

, file = (modelfile <- tempfile ()))

jags.data <- list(stdz.log.inv.metric = stdz.log.inv.metric,

Ntaxa = as.numeric (length (stdz.log.inv.metric)),

stdz.log.trait1 = stdz.log.trait1,

stdz.log.trait2 = stdz.log.trait2,

stdz.log.trait3 = stdz.log.trait3)

inits <- function() list (alpha=rnorm(1), beta1=rnorm(1), beta2=rnorm(1), beta3=rnorm(1), sd=rlnorm(1))

parameters <- c ("alpha", "beta1", “beta2”, “beta3”, "sd", "residual", “predicted”)

n.chains=3

n.iter=10000

n.burnin=2000

out <- jags (data = jags.data, inits = inits, parameters.to.save = parameters, model.file = modelfile, n.chains=n.chains, n.iter=n.iter, n.burnin=n.burnin

**Appendix S3. Criteria followed to filter TRY records for imputation**

Trait records meeting the following criteria were excluded from the imputation process:

1. Altitude > 1,986m (highest point in Victoria; Mount Bogong)
2. Latitude between -10 and 10, below -50, or above 50 degrees
3. Recorded on an experimental design, or horticultural environment
4. Measured on shade
5. Measured on seedlings, saplings or juvenile individuals
6. Collected in habitats described as tropical forest

**Appendix S4. Imputation of missing traits**

Consistent with previous findings (Taugourdeau *et al.*, 2014; Moreno-Martínez *et al.*, 2018), the imputation method that used both phylogenetic relationships and correlations among traits (*Dataset IV – Off-site data and bhpmf imputation*) provided the best estimates for missing SLA and height (Fig. 6c). The addition of trait correlations, however, came at the expense of having to collect information for additional traits (in our case, seed mass). Imputation methods based solely on phylogenetic relationships (*Datasets II* and *III*) were unreliable as they assigned similar trait means and high uncertainty to all species with missing values (Fig. 6a,b). Further investigation of all public SLA and height records from the TRY database revealed a strong overlap of trait values among clades (Fig. S13), in line with previous studies (Lord *et al.*, 1995). Results from our taxonomy-based imputation (*Dataset II*) also showed that most of the trait variability occurs within genera (Fig. S14, sd_sp_), as opposed to at higher taxonomic levels (i.e. families; Fig. S14, sd_g_ and sd_f_). Greater trait variation between congeneric species, rather than among genera, leads to large uncertainty around genus-level trait mean estimates (i.e. wide credibility intervals) and large overlap in trait values among low taxonomic levels (Fig. S13). Biases and gaps in online databases, e.g. common taxa are better represented than rare taxa (Violle *et al.*, 2015; Cornwell *et al.*, 2019), likely contribute to some of our genus-level estimates borrowing information from other clades. In particular, estimation of the within-genus variation for genera represented by few species in the database will borrow information from genera that have more species, and therefore more accurate genus-level mean trait estimates. Ultimately, this has the potential to drive strong shrinkage towards the global mean of the whole dataset.

Validation results for taxonomic, phylogenetic and *bhpmf* imputation methods (Fig. 6) should be interpreted with care, though, since they are based on the availability of TRY records, and not on the “real” (and unknown) trait mean of the species. Despite the apparent improvement in missing trait estimation from taxonomic to phylogenetic to *bhpmf* methods, *Datasets II*, *III* and *IV* showed similarly low correlations with *Dataset I* (*On-site data*) (Figs. 3,4).

The imputation methods we present in this paper voiced existing doubts (Schrodt *et al.*, 2015; Johnson *et al.*, 2020) about the use of point estimates to impute missing trait values, and suggests that the uncertainty around those estimates may need to be incorporated in subsequent analyses.

**REFERENCES**

Cornwell W.K. et al. (2019) *What we (don't) know about global plant diversity.* **Ecography** 42: 1819-31.

Johnson, T.F. et al. (2020) *Handling missing values in trait data*. **Global Ecology and Biogeography** 30: 51-62.

Lord, J. et al. (1995) Seed Size and Phylogeny in Six Temperate Floras: Constraints, Niche Conservatism, and Adaptation**. The American Naturalist** 146: 349-364.

Moreno-Martínez, Á. et al. (2018) *A methodology to derive global maps of leaf traits using remote sensing and climate data*. **Remote Sensing of Environment** 218: 69-88.

Pérez-Harguindeguy N. et al. (2013*) New handbook for standardised measurement of plant functional traits worldwide***. Australian Journal of Botany** 61: 167-234.

R Core Team (2020) R: a language and environment for statistical computing. R Foundation for Statistical Computing, Vienna, Austria. https:// www.R-project.org/

Schrodt, F. et al. (2015) *BHPMF – a hierarchical Bayesian approach to gap-filling and trait prediction for macroecology and functional biogeography*. **Global Ecology and Biogeography** 24: 1510-21.

Taugourdeau, S. et al. (2014*) Filling the gap in functional trait databases: use of ecological hypotheses to replace missing data.* **Ecology and Evolution** 4: 944-58.

Violle, C. et al. (2015) *Trait databases: misuses and precautions*. **Journal of Vegetation Science** 26: 826-27.

Yu-Sung, S., Masanao, Y. (2015) R2jags: Using R to Run 'JAGS'. R package version 0.5-7. https://CRAN.R-project.org/package=R2jags
